# Identification of antibiotic resistance genes and their bacterial hosts in samples collected from wastewater treatment plants in Palestine

**DOI:** 10.1128/spectrum.00028-26

**Published:** 2026-09-18

**Authors:** M. Hroub, S. Ereqat, A. Al-Jawabreh, Z. Abdeen

**Affiliations:** 1 Hebron University, Al-Khalil, Palestine; 2 Biochemistry and Molecular Biology Department, Faculty of Medicine, Al-Quds University 61147, Jerusalem, Palestine; 3 Leishmaniases Research Unit-Jericho, Jericho, Palestine; 4 Arab American University 61287, Jerusalem, Palestine; 5 Al-Quds Public Health Society, Jerusalem, Palestine; Public Health Agency of Canada, Winnipeg, Manitoba, Canada; Security Forces Hospital, Riyadh, Saudi Arabia

**Keywords:** ARGs, ARB, MDR, wastewater treatment, activated sludge, influent, effluent

## Abstract

Wastewater treatment plants are considered disseminators of antibiotic resistance bacteria (ARB) and antibiotic resistance genes (ARGs). In the West Bank, Palestine, there are four working wastewater treatment plants (WWTPs) located in Jenin, Tulkarem, Ramallah (Al-Terah), and Al-Bierh. Most of which have secondary treatment facilities that depend on the activated sludge process. In this study, Illumina high-throughput sequence analysis was used to determine the profile of ARB and ARGs in Al-Bierh and Al-Terah WWTPs. Raw wastewater sample (influent) and secondary treated water sample (effluent) were collected over two seasons, summer and winter. The results showed a higher number of ARB and a wider diversity of ARGs in the winter samples than in the summer samples. The most abundant species found in both seasons and across the samples were *Acinetobacter baumannii* in the Al-Bireh sample, and *Pasteurella multocida* in the Al-Terah sample. Moreover, 138 different ARGs conferring resistance to 12 antibiotic classes were detected. The most abundant ARG was the one resistant to macrolides and tetracyclines. The removal efficiency of the 10 most prevalent ARB and ARGs was high, ranging from 85% to 100% in both treatment plants. In conclusion, the unfiltered antibiotics and ARGs can be taken up by plants and crops and thus pose a public health problem. The abundance of ARGs and their bacterial hosts was found to be higher in winter. Therefore, improving the sanitation and efficiency of treatment systems should be a priority for policymakers to limit the burden of ARB and ARGs in treated wastewater in Palestine.

**IMPORTANCE:**

Untreated wastewater plays a key role in spreading antibiotic-resistant bacteria (ARB) containing antibiotic-resistant genes (ARG) in the environment. The current study characterized the ARB and ARG in two wastewater treatment plants (WWTP) in Jenin and Ramallah, Palestine, in summer and winter in both pre-treated and post-treated water. In winter, ARB were more abundant, and ARGs were highly diverse in both plants alike. *Acinetobacter baumannii*, an extremely drug-resistant bacterium, was found as a dominant bacterium. The study revealed 12 ARG groups based on the antibiotic class in the two WWTPs, with β-lactamases showing the highest number of resistant genes. The efficiency of removal of bacteria in both plants ranged from 85% to 100%.

## INTRODUCTION

Wastewater treatment (WWT) is a method of removing pollutants and contaminants from wastewater (WW). The process consists of biological, chemical, and physical pathways to remove contaminants. After treating wastewater, it could be released safely into the environment. Sludge is a by-product of the treatment process that comes from sewage and needs further processing before use or elimination. It consists of two types, sludge and activated sludge, with both containing a variety of organic and inorganic compounds. The sludge consists of a large percentage of water that comes from liquid wastewater containing a small amount of solid waste. It can be primary, which comes as a result of primary treatment, or secondary, which results from the secondary treatment process. Activated sludge (AS) contains various types of microorganisms that live in harmony together. The diversity of microbes in AS is more than the incoming wastewater (IW) and contains uncultivable types, which do not grow on standard culture media, such as 5% sheep blood agar media, chocolate media, and MacConkey agar. The difference between sewage and sludge is that the former is a suspension of solid and water waste, in contrast to the sludge, which is solid separated from the suspension in a liquid (1). The WW treatment process includes three steps: physicochemical as a primary step, biological as a secondary step, and a tertiary process that executes special methods, such as the advanced oxidative process. The main goal is to obtain high-quality water to be reused in different applications (2). A huge diversity of pathogenic and nonpathogenic microorganisms is found living together in wastewater, sharing metabolic activities to support the growth and multiplication of one another. The wastewater treatment plants (WWTPs) became a hotspot for the presence of microorganisms, especially the antibiotic-resistant organisms that come from humans and animals and are released into sewage through feces, urine, dead bodies, and manure. Moreover, WW that flows from hospitals and farming facilities could be the major source of antibiotic resistance genes (ARGs) and antibiotic-resistant bacteria (ARB) that are released into the environment (3).

The main route of transfer of resistance in the AS occurs by horizontal transfer of ARGs through mobile genetic elements (MGEs), such as plasmids, transposons, bacteriophages, insertion sequences, and integrons (4). There are many ARGs that encode resistance against different antibiotics like tetracyclines (tet), sulfonamides (sul), β-lactams (bla), macrolides (erm), aminoglycosides (aac), fluoroquinolones (fca), colistin (mcr), vancomycin (van), and multidrug (mdr) (5). It was shown that MGEs were abundant in sludge samples, and this may be important for the acquisition and mobility of various ARGs among the bacterial species (6). The spreading of ARGs and ARB in the environment could lead to antibiotic resistance in humans and threaten public health (3).

In the past, culture-based identification methods were confronted with trouble in the analysis of the microbial community from environmental samples because they largely depended on morphology for the identification of microorganisms. Other limitations of this method include the low selectivity of culture media and the wide range of different culture conditions that inhibit or hinder the growth of 99% of the WW bacterial community (7, 8). On the other hand, molecular-based methods have been used for the identification of microorganisms and assessing their biological activity under various conditions. They can specifically determine real-time observation of a microorganism and its metabolic pathway. Therefore, the shotgun metagenomic method can determine the whole genomic DNA in the environmental sample without isolating or culturing each microorganism in the lab (9), in contrast to classical PCR method, which depends solely on specific primers to isolate ARGs and detect their abundance in the bacteria. Besides that, classical PCR is not as useful in detecting novel ARGs as next-generation metagenomic analysis (10). Furthermore, the new next-generation sequencing (metagenomic) approach can detect low-abundance microorganisms by generating hundreds to thousands of sequences and give a clear picture of the microbial community found in the sample (11).

Using metagenomics analysis, this study aimed to profile antibiotic-resistant bacteria and their ARGs isolated from two WWTPs throughout two seasons, summer and winter, in August and February, analyzed from pre- and post-treatment samples, and to assess the removal efficiency of the targeted WWTPs in Palestine. Summer in Ramallah, Palestine, is dry and hot with zero rainfall compared with rainy and cold winter with an average rainfall of 615 mm. The average temperature in summer is approximately 35°C compared with 7°C in winter. The City of Ramallah lies on the latitude line of 31.8829994201660 north and on the longitude line of 35.1926994323730 east.

## MATERIALS AND METHODS

### WWTP description

The water samples were collected from both Al-Bireh and Al-Terah WWTPs, located in Ramallah City. Al-Bireh plant was constructed in 1997 and started functioning in February 2000. It has a capacity of 6,000 m^3^/day of treated wastewater and serves about 50,000 people in Al-Bireh town. This WWTP depends on the conventional activated sludge method as a secondary treatment process, including a low-loaded activated sludge stage, which, besides the removal of carbonaceous compounds, performs oxidation of nitrogen compounds (nitrification). The treatment process is performed in a unit composed of a mixing and distribution chamber for aeration tank feeding without the involvement of tertiary treatment process. However, the effluent output of this plant is released into the wadies (Fig. S1a). Al-Terah plant, located in Al-Terah town, serves 47,500 people. The capacity of this plant is 1,370 m^3^/day. It depends on two aeration tanks to treat wastewater. However, the treated water is deposited in Wadi Bitunia (12) (Fig. S1b).

### Sampling

Two different types of samples from both WWTPs were collected using sterile 500-mL bottles (Duran, Inc., USA). From each WWTP, one raw (influent) and one treated (effluent) sample were collected in two different seasons: winter (February) and summer (August). The composite sampling involved taking equal subsamples every 5 m in sterile bottles to finally have the whole sample to ensure representativeness, then transported within 48 h in a cool box to the laboratory for DNA extraction.

### Samples preparation

Influent samples (500 mL) were first divided equally into ten samples using 50-mL sterile Falcon tubes, then centrifuged at maximum speed, about 3,220 g, for 20 min in a 5810 R centrifuge (Eppendorf, Germany) to obtain the pellet. The pellets were reconstituted with absolute ethanol in a ratio of 1:2 and kept at −20°C to be used for DNA extraction (13). The 500-mL effluent samples were filtered using a polyvinylidene difluoride (PVDF) membrane filter (Stericup 250 mL, Durapore, 0.45-μm PVDF). The membrane was then carefully removed using sterile scissors and forceps, transferred to sterile tubes, and kept at −20°C to be used for DNA extraction.

### DNA extraction and concentration

#### Influent samples

The 50-mL frozen samples were thawed, homogenized separately by vortex mixer, and finally the ten tubes collected into one tube. For DNA extraction, QIAamp DNA Mini Kit (Qiagen GmbH, Germany) was used according to the manufacturer’s instructions for bacterial DNA extraction protocols, except in the final step, in which 50 μL of elution buffer was added instead of 200 μL to obtain a final extract volume of 50 μL.

#### Effluent samples

The filter papers were thawed, carefully removed from the tube, and cut into small pieces using sterile scissors. The DNA was extracted from filter papers using the same kit and procedures used for influent samples. The concentration of DNA samples was measured using the Qubit v4 (Invitrogen, USA) machine. DNA concentration was adjusted to 0.4 ng/μL to be accepted for library preparation.

#### Library preparation

For metagenomic analysis, the DNA library was prepared using the Nextera XT DNA library preparation kit (Illumina, USA). Briefly, the gDNA was normalized to have 5 μL of DNA. Tagmentation was done using 5 μL of Tagmentation buffer (Amplicon Tagment Mix) with a special Tagmentation mixture that contains transposomes and then incubated at 55°C for 5 min. The reaction was kept at 10°C to stop the action of transposomes. Then, 5 μL of Neutralize Tagment Buffer (NT) was added to the mix and incubated for five minutes at room temperature. The final volume was 25 μL for the next step. An enrichment process was conducted in which two different index adapters were added (5 μL each) at both ends of the DNA fragment. To attach those index adapters to the DNA, a 15 μL of Nextera PCR master mix was added to 35 μL of tagmented DNA to a final volume of 50 μL. The reaction mixture was subjected to thermal cycling as follows: 72°C for 3 min, 95°C for 30 s, then 12 cycles of 95°C for 10 s, 55°C for 30 s, 72°C for 30 s, then 72°C for 5 min, and finally held at 10°C. The obtained DNA library with fragments between 300 and 500bp, which is double the size of the selected DNA sequence, was then purified with 1× AMPure XP beads (Beckman Coulter, Brea, CA, USA). The concentration of the prepared DNA library was measured using the Qubit v4 (Invitrogen, USA) machine. The quality of the library was evaluated by the Tapestation machine (Agilent, CA, USA), and the library was normalized to 4 nmol. Sequencing was done using NextSeq 500/550 High Output Kit v2.5 (150 Cycles). The sequencing was done as a single read with a length of 150 bp. Output sequencing data were received as fastq files.

### Bioinformatics analysis

#### Analysis workflow

First, the fastq files were converted into FASTA format using the FASTQ to FASTA converter from the FASTX toolkit command on the online Galaxy software (www.usegalaxy.org). Then, the sequence files (FASTA) were uploaded to the https://usegalaxy.org.au/ platform. The annotation process was done using the ABRicate command, which performs mass screening of contigs for antimicrobial and virulence genes. The output results of this command included the related reference sequence, which was indicated by the GenBank accession number. The accession number, accompanied by each ARG from the previous Galaxy workflow analysis, was BLAST searched (www.blast.ncbi.nlm.nih.gov) to identify the bacterial host. Each organism with an identity and query cover above 97% was included (14). After downloading the results file of the ABRicate, the statistical package SPSS v29 (IBM Corporation, USA) was used to measure the frequency of each ARGs of the influent and effluent samples in the two different seasons. Then, “Cross Tab” command as applied to link the accession number to different types of ARGs (Table S1). Adaptors, sequencing artifacts, and low complexity reads were removed as part of quality filtering using BLAST search tool and Galaxy workflow analysis.

##### ARGs analysis

AMR gene family, drug class, and resistance mechanisms for each gene were analyzed using the Comprehensive Antibiotic Resistance Database (CARD, https://card.mcmaster.ca/), which is a bioinformatics database of resistance genes, their products, and associated phenotypes. The “Analyze” command on the CARD main page was chosen, then the reference gene identifier (RGI) was selected to search for resistant genes using the GenBank accession numbers obtained from the previous Galaxy workflow analysis.

### Quality control and assembly

Quality control for each sample was performed using FastQC (version 0.12.1) on mapped reads in BAM format. Raw reads were first processed with Trimmomatic (Galaxy version 0.36.6), and the resulting data set was subsequently used as input for Cutadapt (Galaxy version 5.2). The output from Cutadapt was then assembled using the single-end read assembler SPAdes (Galaxy version 4.2.0). The resulting contigs were evaluated with Quast (Galaxy version 5.3.0), which provided quality control metrics for each sample. The average total depth was calculated using the Calculate contig depths for MetaBAT2 tool (Galaxy version 2.17), with filtered BAM reads as input.

### Removal efficiency

The removal efficiency of ARGs was calculated by the following equation described elsewhere (15), which was based on calculating the raw number of reads for ARB and ARG.


$$\%\;Removal\;Efficiency=\frac{Influent\;frquency}{Influent\;freq-Effluent\;freq}\;x\;100\%$$

## RESULTS

Our study utilized a metagenomic sequencing approach to examine the ARGs present in influent and effluent samples collected from both Al-Bireh and Al-Terah plants over two different seasons. In winter, the total number of reads was higher in Al-Terah WWTP than in Al-Bireh in the influent and effluent samples. In summer, Al-Bireh had more reads than Al-Terah in the influent and effluent samples (Table 1).

**TABLE 1:** The overall metagenomics characteristics in the two WWTPs, Al-Bireh and Al-Terah, in winter and summer

	Al-Bireh WWTP		Al-Terah WWTP	
	Winter	Summer	Winter	Summer
No. of reads, influent	3.7 × 10^6^	0.37 × 10^6^	5.09 × 10^6^	0.031 × 10^6^
No. of reads, effluent	0.095 × 10^6^	0.26 × 10^6^	0.32 × 10^6^	0.022 × 10^6^
No. of ARGs, Influent	400	253	305	162
Prevalent ten, %	44	38	51	56
ARGs	(*msr(E)*, *mph(E)*, *tet(Q)*, *cfxA2*, *tet(39)*, sul1, *erm(F)*, *mph(A)*, *tet(Q)*, *aph(6)-Id*)	(*msr(E)*, *mph(E)*, *mph(A)*, *sul1*, *aph(6)-Id*, *tet(39)*, *cfxA2*, *ant(3*''*)-Ia*, *cmlA1*, and *msr(D)*)	(*msr(E), mph(E), sul1, tet(39), tet(Q), mph(A), qnrS2, aph(6)-Id, cmlA1,* and *cfxA2*)	*(msr(E), mph(E), tet(39), tet(Q), sul1, sul2, mef(C), cfxA2, aph(3*''*)-Ib,* and *mef(A)*)
Antibiotics, influent	Macrolide, tetracycline, beta-lactamase, sulfonamide, streptogramin, aminoglycoside	Macrolide, tetracycline, aminoglycoside, sulfonamide trimethoprim	Macrolide, sulfonamide, tetracycline, aminoglycoside, chloramphenicol/florfenicol, beta-lactamase, quinolone	Macrolide, sulfonamide, tetracycline, aminoglycoside, beta-lactamase
No. ARGs, effluent	88	72	97	52
Prevalent ten, %	67	51	62	66
ARG subtypes	*msr(E), mph(E), tet(39), sul1, sul2, erm(F), ant(3*″*)-Ia, oqxB_, mef(C),* and *mph(G)*	*msr(E), mph(E), mph(A), sul1, ere(A), tet(C), cfxA2, ant(3*″*)-Ia, blaOXA-10, blaLCR-1*	*msr(E), oqxB, mph(E), sul1, ere(D), sul2, tet(39), erm(F), ant(3*″*)-Ia,* and *floR*	*msr(E), mph(E), sul1, ant(3*″*)-Ia, erm(F), qepA1, aadA10, aadA2, dfrA1, erm(35)*
Antibiotics, effluent	Macrolide, tetracycline, sulfonamide, streptogramin, aminoglycoside	Macrolide, betalactamase, sulfonamide tetracycline, aminoglycoside	Macrolide, tetracyline, aminoglycoside, chloramphenicol/florfenicol, sulfomamide	Macrolide, sulfonamide, aminoglycoside

### ARG abundance

**Fig 1:**
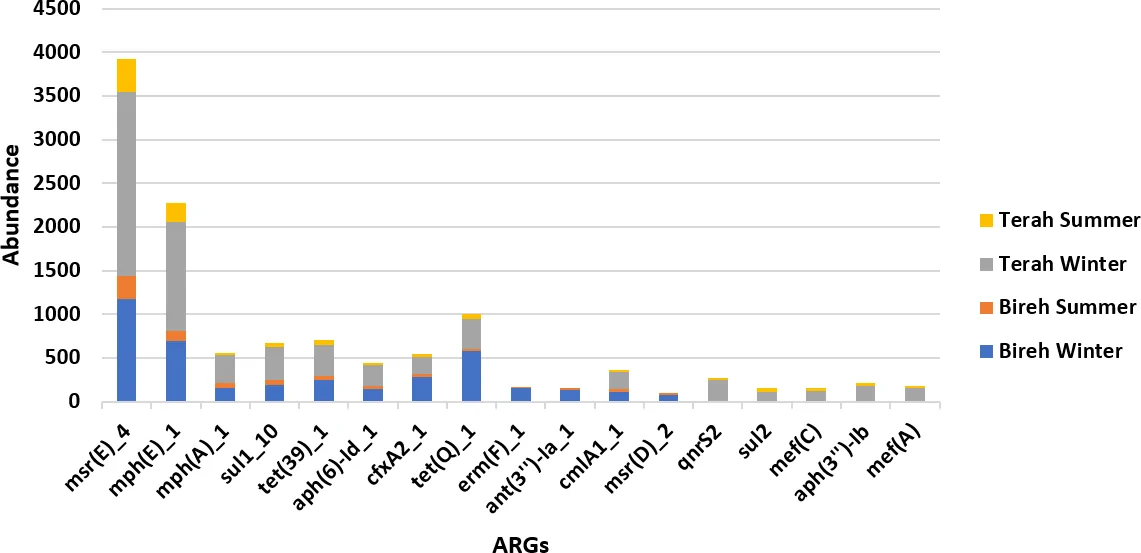
Difference in abundance (no. of reads) of ARGs between winter and summer found in influent samples taken from Al-Bireh and Al-Terah WWTPs.

In winter, the number of ARG subtypes in influent samples from Al-Bireh WWTP was 400. The 10 most prevalent ARGs comprised 44% of the total ARGs and include in order of high abundance (*msr(E), mph(E), tet(Q), cfxA2, tet(39), sul1, erm(F), mph(A), tet(Q),* and *aph(6)-Id*) (Fig. 1; Table 1). The ARGs were found to be resistant against the antimicrobial drug families, macrolide, tetracycline, followed by Beta-lactamase, sulfonamide, streptogramin, and aminoglycoside. In influent samples from Al-Terah WWTP, 305 ARGs subtypes were explored, showing the highest ten prevalent subtypes comprising 51% of the total, namely (*msr(E), mph(E), sul1, tet(39), tet(Q), mph(A), qnrS2, aph(6)-Id, cmlA1,* and *cfxA2*) (Fig. 1; Table 1). The ARGs were resistant to antibiotic families similar to those existing in Al-Bireh WWTP, with a few differences, like quinolone, found in Al-Terah WWTP only. While in summer, 253 ARGs subtypes were found in the influent samples from Al-Bireh WWTP, with the leading 10 ARGs accounting for 38%. These ARGs included (*msr(E), mph(E), mph(A), sul1, aph(6)-Id, tet(39), cfxA2, ant(3*″*)-Ia, cmlA1,* and *msr(D)*). Like in winter samples, the most abundant antimicrobial drug class was macrolide resistance, followed by tetracycline resistance genes, aminoglycoside, sulfonamide, and trimethoprim. While in Al-Terah plant, 162 ARGs were found, and the leading 10 comprised 56%, which were *msr(E), mph(E), tet(39), tet(Q), sul1, sul2, mef(C), cfxA2, aph(3*″*)-Ib,* and *mef(A)*. The resistance gene families were the same as in the Al-Bireh sample.

**Fig 2:**
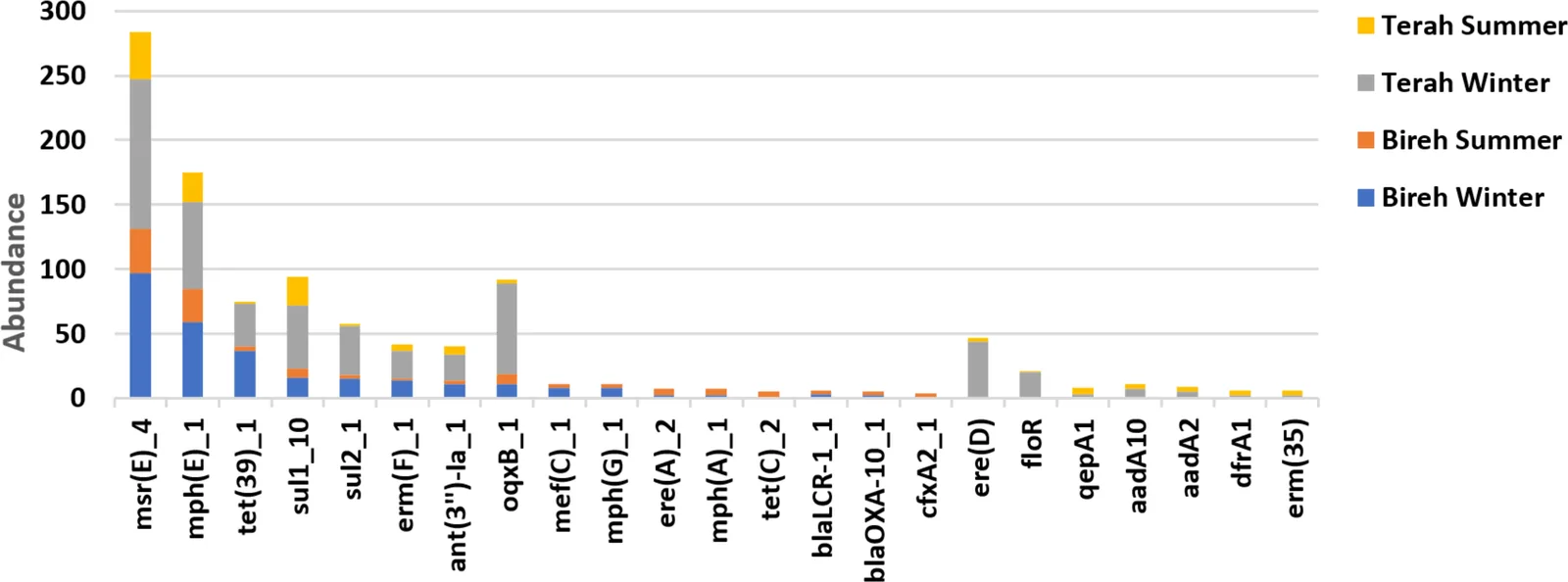
Differences in abundance (no. of reads) of ARGs in winter compared with summer in effluent samples from Al-Bireh and Al-Terah WWTPs.

In winter, the effluent sample from Al-Bireh WWTP, 88 ARGs subtypes with *msr(E), mph(E), tet(39), sul1, sul2, erm(F), ant(3*″*)-Ia, oqxB, mef(C),* and *mph(G)* comprising 67% of the total ARGs (Fig. 2, Table 1). The most abundant ARG families were as in the influent sample. But, in the Al-Terah plant, there were 97 ARGs subtypes with *msr(E), oqxB, mph(E), sul1, ere(D), sul2, tet(39), erm(F), ant(3*″*)-Ia,* and *floR*, comprising 62%. The ARGs were resistant to antibiotic families similar to those found in influent samples. On the other hand, in summer, the effluent samples taken from Al-Bireh and Al-Terah WWTPs revealed 72 and 52 ARGs subtypes, respectively. The 10 most prevalent ARGs constituted 51% and 66% of the total in the Al-Bireh and Al-Terah samples, respectively (Fig. 2, Table 1). The most abundant ARGs in the influent sample were resistant to macrolide, sulfonamide, tetracycline, and aminoglycoside antibiotic classes (Table S2).

### Identification of ARGs bacterial hosts and removal efficiency

In winter, 53 and 100 different bacterial species were found in influent samples from Al-Bireh and Al-Terah WWTPs, respectively. These data were retrieved by BLAST analysis with a query cover of over 97% that included the high potential bacteria that are known to harbor ARGs. Ten bacterial species were found to comprise 74% and 77% of the total species abundance in Al-Bireh and Al-Terah plants, respectively. Regarding phyla frequency, the most abundant phyla were *Pseudomonadota* (67.8%), *Firmicutes* (8.4%), and *Bacteroidota* (6.3%).

Uncultivable bacteria represented 11.3% and 11% in Al-Bireh and Al-Terah WWTPs, respectively. According to bacterial genera and species, *Acinetobacter baumannii* had the highest frequency in Al-Bireh WWTP compared with *Pasteurella multocida* in Al-Terah WWTP. While in summer, the influent samples from Al-Bireh WWTP contained 30 different bacterial species, with the most prevalent 10 comprising 81% of the total bacterial species. However, in the Al-Terah influent sample, 68 species were found, with the most frequent ten comprising 76% of the total. At the phylum level, the most abundant phyla were *Pseudomonadota* (72%), *Firmicutes* (12%–13%), uncultivable bacteria (10%–11%), and *Bacteroidota* (2%) in the influent samples from Al-Bireh and Al-Terah samples.

On the other hand, winter effluent samples, only eight bacterial species were found in Al-Bireh and 52 in Al-Terah WWTPs. The most common phyla were *Pseudomonadota* (63.55%) and *Bacteroidota* (6.3%), while uncultivable bacteria represented 1.27% in both Al-Bireh and Al-Terah plants, respectively. In the Al-Terah plant, *Firmicutes* (5%) and Actinobacteria (7%) were additionally found. However, in summer, the effluent sample from Al-Bireh WWTP contained only two bacterial species: *A. baumannii* and *K. pneumoniae*, belonging to the phylum *Pseudomonadota*. In contrast, the Al-Terah effluent sample was shown to contain 21 bacterial species. The dominant phyla were *Pseudomonadota* (58%), followed by *Firmicutes* (13%), *Bacteroidota* (3%), and *Actinobacteria* (2%), whereas uncultivable bacteria comprised 22% (Table S2).

In the samples from Al-Bireh WWTP, some of the bacteria, such as *Prevotella* species and *Streptococcus pneumoniae*, were cleared from the effluent sample, and thus the removal efficiency was considered 100%. The removal efficiency for the other bacteria was still high, ranging from 67% to 100%. While in Al-Terah WWTP samples, only *Acinetobacter johnsonii* has been cleared completely, with the removal efficiency ranging from 63 to 100%. (Table S1). The removal efficiency of ARGs ranged from 85% to 100% in Al-Bireh WWTP and 87%–100% in Al-Terah WWTP.

There are 12 ARG classification groups. The most diverse one that comes with different resistance genes is beta-lactamase, with 34 different genes. Followed by tetracycline and aminoglycoside, which have 20 and 18 genes, respectively. The other groups were ordered according to the number of genes. (Table S2).

## DISCUSSION

As antibiotics have been used for many years to eliminate bacteria, a wide range of these bacteria have acquired resistance to antibiotics through horizontal and vertical gene transfer (HGT) and mutations (16). This has introduced antibiotic ARB and ARG on a large scale into the environment, becoming an emerging global health problem (3, 17, 18). This gives the reason for estimating the level of ARGs in the activated sludge that ends up in the environment. The study aimed to document the presence of various types of ARB and ARGs in raw influent wastewater (WW) and treated effluent WW collected in two different seasons, winter and summer, from two WWTPs. The sources of raw wastewater that flow to those plants are houses, slaughterhouses, and different hospitals in the area. In this study, a difference was observed in the amount of bacterial DNA, as represented by the number of bacterial reads, between influent and effluent samples across the two seasons. The decreased number of reads from thousands of reads prior to treatment to a couple of hundred post-treatment may reflect, to some extent, the efficiency of the treatment plant. However, the remaining amount of ARG in effluent can still spread in the environment with high-risk potential (19). On the other hand, the reason for the high amount of bacterial DNA in winter rather than summer is due to rainwater that carries most of the microorganisms in the sewer pipeline and goes directly to plants. Moreover, the prevalence of bacterial infection is usually higher in winter than summer, especially among vulnerable people with weak immunity, such as children and the elderly, who may overuse broad-spectrum antibiotics and thus develop resistant bacteria (19, 20). Also, most bacteria, including ARB, that cause gastrointestinal, respiratory tract, and urinary tract infections are shed into the sewage system. In addition, the overuse of antibiotics results in the excretion of unmetabolized antibiotics into the environment, which, with the different pressures of pollutants, causes the spread of ARGs among bacterial communities in the environment (21). In the Al-Bireh plant, the treatment process depends basically on activated sludge, which uses bacteria to degrade the contaminants and thus the microbial community. While in Al-Terah, it depends mainly on two aeration lagoons to treat water.

**Fig 3:**
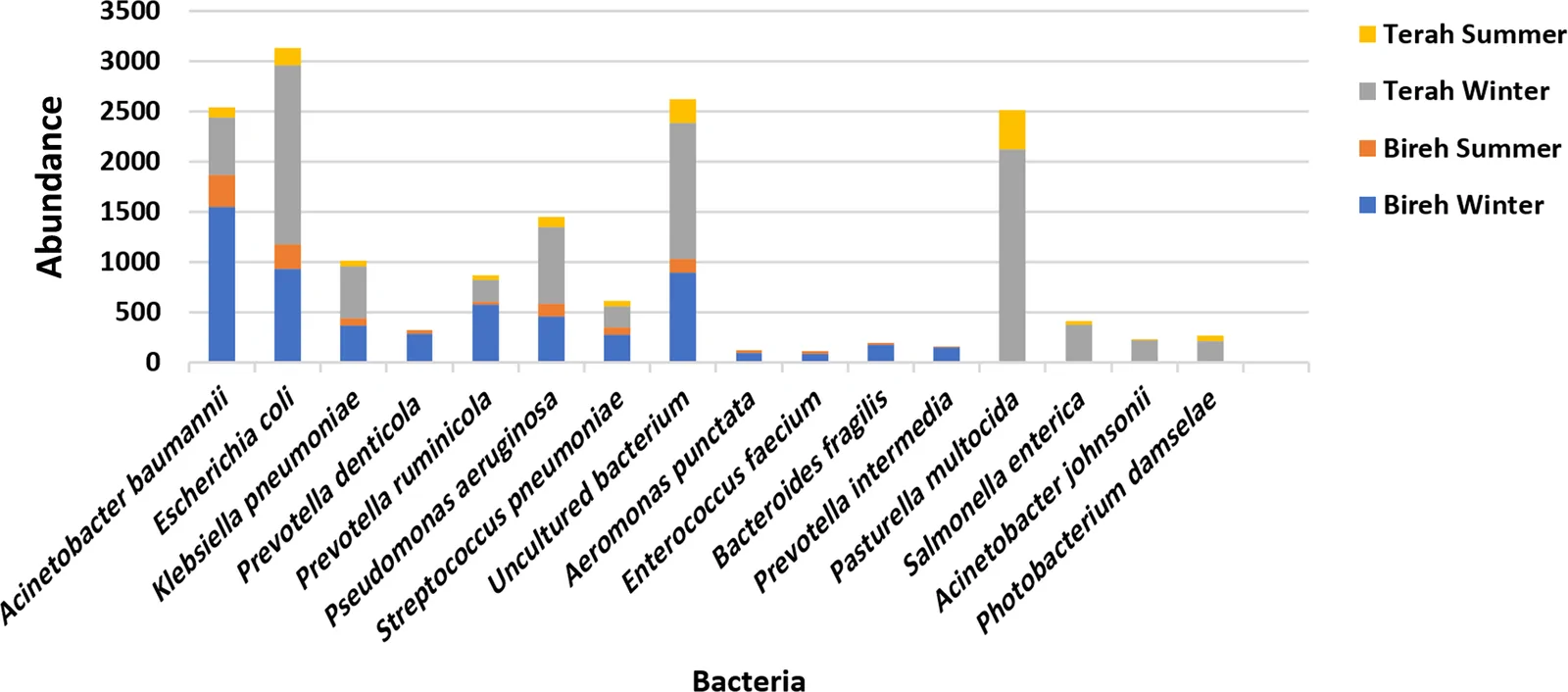
Differences in abundance (number of reads in ppm) of major ARB species between winter and summer in influent samples taken from Al-Bireh and Al-Terah WWTPs.

**Fig 4:**
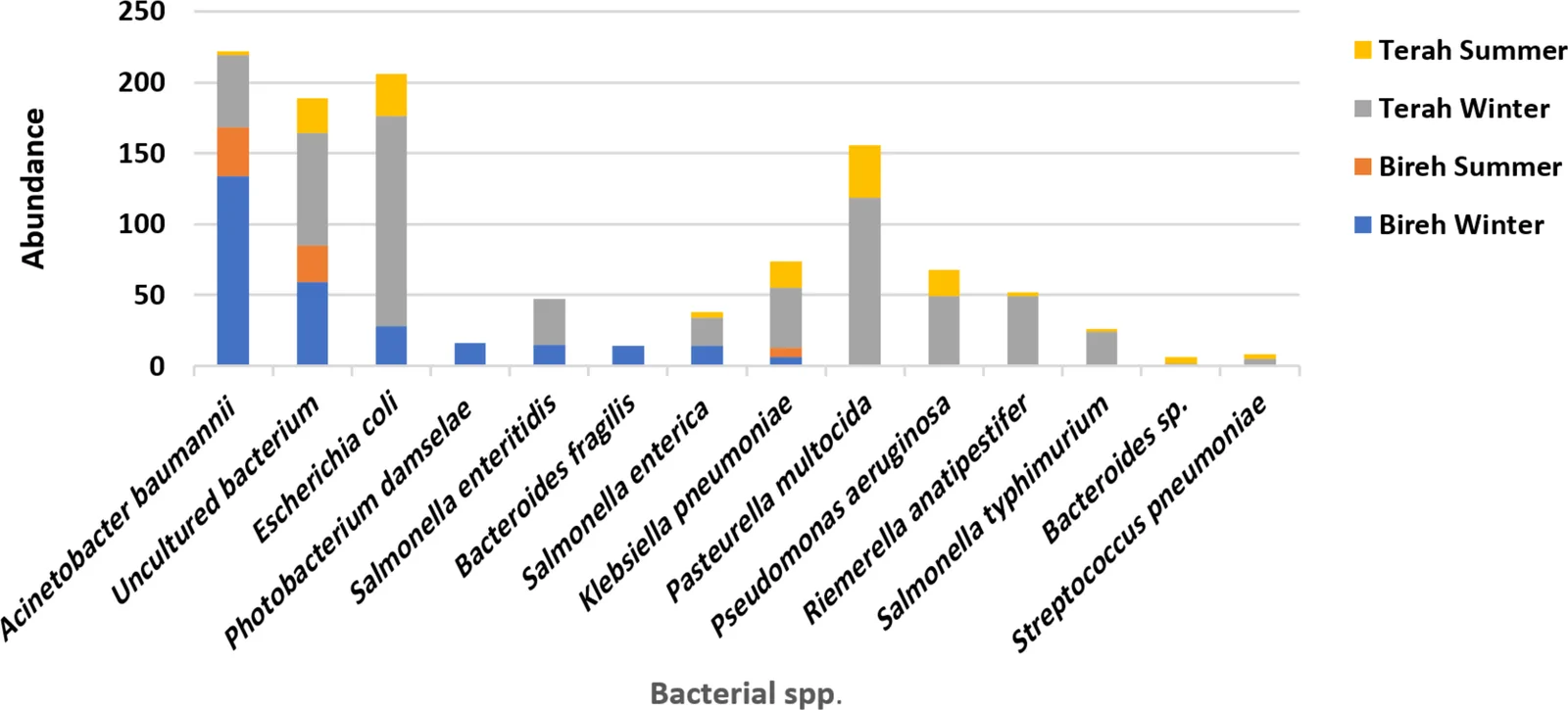
Differences in abundance of the major bacterial species (No. of reads in ppm) between winter and summer in effluent samples from Al-Bireh and Al-Terah WWTPs.

In winter, more bacterial species (54,100) were detected in the influent samples than in summer samples (3,068) taken from both Al-Bireh and Al-Terah WWTPs, respectively. Most of the bacteria detected in the influent and effluent samples were gram-negative bacteria, including *Salmonella* enterica and *Pseudomonas aeruginosa* (Fig. 3 and 4), the WHO priority pathogens (22). The detected ARB are the most common causing disease and may survive at a wide range of temperatures and pH, such as *Pseudomonas aeruginosa*, with a growth range between 4°C and 42°C and pH of 4.5 to 9.0 (23). He and colleagues (24) showed that the amount and diversity of bacteria in winter were more than in summer, which is explained by increasing the biomass of the reactor to maintain the effectiveness of wastewater treatment under cold temperatures, besides the increase of antibiotic use during winter, which may increase the number of resistant bacteria (25). At the phylum level, the most abundant ARB found in both seasons were *Pseudomonadota*, followed by *Firmicutes* and *Bacteroidota*. Of the *Pseudomonadota*, several significant species were identified, such as *A. baumannii*, *E. coli*, *P. multocida*, and *K. pneumonia*. However, these species were significantly more frequent in winter than in summer. Our results are consistent with several other studies (8, 26, 27) and inconsistent with Ju et al. study, which reported that the most abundant phylum in the influent sample was *Firmicutes*, followed by *Pseudomonadota*, *Actinobacteria*, and *Bacteroidota* (28). Regarding genera frequencies, our results agreed with those reported by other studies (29), which showed that the most abundant bacterial genus across the samples was *Acinetobacter*, and disagreed with other results, stating that the most frequent genera were *Bacteroides, Escherichia,* and *Streptococcus* (29). Ahmed et al. reported that the most dominant bacterial genera in raw wastewater samples were *Pseudomonas*, followed by *Arcobacter* and *Bacteroides* (8). In our study, all the bacterial species were found to match the pattern of human pathogens, which cause different infections in the respiratory tract, gastrointestinal, and urinary tracts (30).

The effluent samples contained eight and 52 species in the winter sample compared with 3 and 21 species in the summer samples from Al-Bireh and Al-Terah, respectively, which runs parallel to the distribution of ARGs. Most of them belonged to the bacterial phyla *Pseudomonadota*, followed by *Bacteroidota*, with lower relative abundance than the influent samples. Our results, inconsistent with another study (31), showed that the most abundant phylum, *Pseudomonadota,* was less frequent in winter than in summer. In the Al-Bireh sample, *Acinetobacter baumannii*, a commonly known multidrug-resistant (MDR) *Proteobacteria* responsible for hospital-acquired infection (HAI), had the highest frequency in both seasons, while other studies showed that *Pseudomonas aeruginosa*, another MDR-HAI bacteria, was the most frequent (8, 29, 32). In contrast, in Al-Terah samples, *Pasteurella multocida*, an animal-related opportunistic commensal *Pseudomonadota*, was found at high frequency.

There were also some bacteria explored that pose a significant health impact, but not in the top 10 bacteria. *Staphylococcus aureus* is an opportunistic pathogen that causes infection. Methicillin-resistant *Staphylococcus aureus* (MRSA) is the most significant type because it harbors different ARGs. *S. aureus* harbors different ARGs like *msr(A), Inu(B),* and *tet(M)*, similar to our finding (33, 34).

In this study, we noticed that most of the bacteria were efficiently removed after treatment, which could be attributed to the high performance of plants, especially in the Al-Bireh than Al-Terah plant. Al-Bireh WWTP is based on using an aeration tank and activated sludge as a secondary process under the effect of oxygen, and the other uses an aeration lagoon, which depends on the oxygen pumped and circulated to build aerobic conditions to treat water. Moreover, it relies on a sedimentation process in which the pathogens are adsorbed in the biosolid phase (8). A dramatic decrease in anaerobic bacteria was noted after treatment, which may be due to shifting from anaerobic to aerobic conditions, replacing the anaerobic bacteria with facultative anaerobic ones with the aid of low temperature, and thus bacteria that live under oxygen conditions will proliferate and be retained in the plant (35).

In the influent samples collected from Al-Bireh and Al-Terah, 400,305 ARGs subtypes were found in the winter samples compared with 253,162 ARGs subtypes found in summer. On the other hand, the effluent samples from the two plants, Al-Bireh and Al-Terah, contained effectively fewer ARGs than in the influent samples, with higher abundance of ARGs in summer (88 and 97, respectively) than in winter (77 and 52, respectively) (Table 1; Table S2). The 1.6-fold ARG increase in winter could be explained by the intensive use of antibiotics (36). However, a few studies showed that temperature differences between seasons have very little or no impact on ARG abundance (37, 38). Our study showed that the most abundant resistance genes in the two were *msr(E)* and *mph(E)*, and to a lesser degree *tet(Q)*. These genes were reported as macrolide resistance genes, especially to erythromycin antibiotics, including azithromycin, clarithromycin, and spiramycin (Fig. 3 and 4), which is in congruence with other studies (29, 36, 39). Several studies revealed different patterns of predominant resistant genes, such as *tet_Q, tetA, tetX* (tetracycline), *ermB* (erythromycin), *sul1* (sulfonamide), *blaCTX-M* (cefotaxime), and *mecA* (methicillin) (8, 40). The discrepancy in ARG profiles between studies can be explained by the different wastewater sources and treatment processes of WWTPs, the class of antibiotics consumed by the served community, and the completeness of ARG databases that rely on the depth of metagenomic sequencing (41). The main study limitation was the absence of multiple copies of each sample, duplicate or triplicate, which could have improved the accuracy and precision of results by adopting a statistical means of results rather than a single result. In addition, the absence of baseline number of abundance (no. of reads) of bacteria and ARGs in both winter and summer, as well as influent and effluent, may have led to the systematic exaggeration of abundance.

### Conclusion

Metagenomic shotgun analysis has an advantage over classical PCR analysis in determining uncultivable bacteria and detecting novel ARGs without targeting specific bacterial genes. In this study, various types of pathogenic bacteria were found, such as *Acinetobacter baumannii*. The abundance of ARGs and related bacterial hosts was found to be higher in February than in August. The removal efficiency was high; however, our concern is the proliferation and dissemination of the remaining unfiltered ARB and ARGs into the environment, which will pollute the ground and freshwater, soil, as well as the plants and crops, and thus pose a public health problem. Therefore, we recommend increasing public awareness about the effect of wastewater containing high amounts of pathogens on human health and the environment. Moreover, improving the sanitation and treatment systems should be a priority for policymakers to limit the burden of ARB and ARGs in treated wastewater in Palestine.

## Supplementary Material

Reviewer comments

## SUPPLEMENTAL MATERIAL

Table S1 spectrum.00028-26-s0001.xlsx The removal efficiency of the ten most prevalent ARGs.

Table S2 spectrum.00028-26-s0002.xlsx The ARGs and bacteria found in the WWTP in summer and winter.

Fig. S1 spectrum.00028-26-s0003.docx (a) The location site of Al-Bireh wastewater treatment plant (AWWTP), north-east of Ramallah city in Palestine (1). (b) A Google Earth map of Al-Tereh WWTP, north-west of Ramallah City in Palestine.

Table S1 legend spectrum.00028-26-s0004.docx The removal efficiency of the ten most prevalent ARGs.

Table S2 legend spectrum.00028-26-s0005.docx The ARGs and bacteria in the the two WWTP in summer and winter.

OPEN PEER REVIEW reviewer-comments.pdf An accounting of the reviewer comments and feedback.

ASM does not own the copyrights to Supplemental Material that may be linked to, or accessed through, an article. The authors have granted ASM a non-exclusive, world-wide license to publish the Supplemental Material files. Please contact the corresponding author directly for reuse.

## References

[1] Poblete IBS, Araujo O de QF, de Medeiros JL. 2022. Sewage-water treatment and sewage-sludge management with power production as bioenergy with carbon capture system: a review. Processes 10:788. doi:10.3390/pr10040788

[2] Fernandes J, Ramísio PJ, Puga H. 2024. A comprehensive review on various phases of wastewater technologies: trends and future perspectives. Eng 5:2633–2661. doi:10.3390/eng5040138

[3] Garcia-Torné M, Falcó I, Borrell X, Bautista A, Mazigh R, Aznar R, Sánchez G, Farré M, Llorca M. 2025. Comprehensive study of antibiotics and antibiotic resistance genes in wastewater and impacted mediterranean water environments. Antibiotics (Basel) 14:341. doi:10.3390/antibiotics14040341 PMC12024384 40298490

[4] Yu Z, Liu Z, Sun L, Dong C, Jin Y, Hu B, Cheng D. 2024. Mobile genetic elements mediate the cross-media transmission of antibiotic resistance genes from pig farms and their risks. Science of The Total Environment 926:172115. doi:10.1016/j.scitotenv.2024.172115 38569972

[5] Jian Z, Zeng L, Xu T, Sun S, Yan S, Yang L, Huang Y, Jia J, Dou T. 2021. Antibiotic resistance genes in bacteria: occurrence, spread, and control. J Basic Microbiol 61:1049–1070. doi:10.1002/jobm.202100201 34651331

[6] Guo J, Li J, Chen H, Bond PL, Yuan Z. 2017. Metagenomic analysis reveals wastewater treatment plants as hotspots of antibiotic resistance genes and mobile genetic elements. Water Res 123:468–478. doi:10.1016/j.watres.2017.07.002 28689130

[7] Hladilek MD, Gaines KF, Novak JM, Collard DA, Johnson DB, Canam T. 2016. Microbial community structure of a freshwater system receiving wastewater effluent. Environ Monit Assess 188:626. doi:10.1007/s10661-016-5630-7 27761851

[8] Ahmed W, Staley C, Sidhu J, Sadowsky M, Toze S. 2017. Amplicon-based profiling of bacteria in raw and secondary treated wastewater from treatment plants across Australia. Appl Microbiol Biotechnol 101:1253–1266. doi:10.1007/s00253-016-7959-9 27838840

[9] Chen Y, Fan LC, Chai YH, Xu JF. 2022. Advantages and challenges of metagenomic sequencing for the diagnosis of pulmonary infectious diseases. Clin Respir J 16:646–656. doi:10.1111/crj.13538 PMC9527156 36068680

[10] Schmieder R, Edwards R. 2012. Insights into antibiotic resistance through metagenomic approaches. Future Microbiol 7:73–89. doi:10.2217/fmb.11.135 22191448

[11] Ahn Y, Choi J. 2016. Bacterial communities and antibiotic resistance communities in a full-scale hospital wastewater treatment plant by high-throughput pyrosequencing. Water (Basel) 8:580. doi:10.3390/w8120580

[12] Mogheir Y, Abu Hujair T, Zomlot Z, Ahmed A, Fatta D. 2005. Treated wastewater reuse in Palestine. International Conference “Water: Values and Rights”; Ramallah, Palestine

[13] Ma L, Li A-D, Yin X-L, Zhang T. 2017. The prevalence of integrons as the carrier of antibiotic resistance genes in natural and man-made environments. Environ Sci Technol 51:5721–5728. doi:10.1021/acs.est.6b05887 28426231

[14] Ravi A, Ereqat S, Al-Jawabreh A, Abdeen Z, Abu Shamma O, Hall H, Pallen MJ, Nasereddin A. 2019. Metagenomic profiling of ticks: Identification of novel rickettsial genomes and detection of tick-borne canine parvovirus. PLoS Negl Trop Dis 13:e0006805. doi:10.1371/journal.pntd.0006805 PMC6347332 30640905

[15] Freeman CN, Scriver L, Neudorf KD, Truelstrup Hansen L, Jamieson RC, Yost CK. 2018. Antimicrobial resistance gene surveillance in the receiving waters of an upgraded wastewater treatment plant. FACETS 3:128–138. doi:10.1139/facets-2017-0085

[16] Amarasiri M, Takezawa T, Malla B, Furukawa T, Sherchand JB, Haramoto E, Sei K. 2022. Prevalence of antibiotic resistance genes in drinking and environmental water sources of the Kathmandu Valley, Nepal. Front Microbiol 13:894014. doi:10.3389/fmicb.2022.894014 PMC9441849 36071971

[17] Ying GG, He LY, Ying AJ, Zhang QQ, Liu YS, Zhao JL. 2017. China must reduce its antibiotic use. Environ Sci Technol 51:1072–1073. doi:10.1021/acs.est.6b06424 28094517

[18] Li P, Wu Y, He Y, Zhang B, Huang Y, Yuan Q, Chen Y. 2020. Occurrence and fate of antibiotic residues and antibiotic resistance genes in a reservoir with ecological purification facilities for drinking water sources. Science of The Total Environment 707:135276. doi:10.1016/j.scitotenv.2019.135276 31864005

[19] Luo C, Zhang T, Mustafa MF, Li M, Xu S. 2025. Removal efficiency of ARGs in different wastewater treatment plants and their potential risks in effluent. npj Clean Water 8:1–9. doi:10.1038/s41545-025-00456-4

[20] Bonanno Ferraro G, Bonomo C, Brandtner D, Mancini P, Veneri C, Briancesco R, Coccia AM, Lucentini L, Suffredini E, Bongiorno D, Musso N, Stefani S, La Rosa G. 2024. Characterisation of microbial communities and quantification of antibiotic resistance genes in Italian wastewater treatment plants using 16S rRNA sequencing and digital PCR. Science of The Total Environment 933:173217. doi:10.1016/j.scitotenv.2024.173217 38750766

[21] Sharma VK, Johnson N, Cizmas L, McDonald TJ, Kim H. 2016. A review of the influence of treatment strategies on antibiotic resistant bacteria and antibiotic resistance genes. Chemosphere 150:702–714. doi:10.1016/j.chemosphere.2015.12.084 26775188

[22] Drane K, Sheehan M, Whelan A, Ariel E, Kinobe R. 2024. The role of wastewater treatment plants in dissemination of antibiotic resistance: source, measurement, removal and risk assessment. Antibiotics 13:668. doi:10.3390/antibiotics13070668 PMC11274174 39061350

[23] Diggle SP, Whiteley M. 2020. Microbe profile: *Pseudomonas aeruginosa*: opportunistic pathogen and lab rat. Microbiology (Reading) 166:30–33. doi:10.1099/mic.0.000860 PMC7273324 31597590

[24] He Y, Li K-X, Wang J-W, Wang W, Fan P-C, Chen H-H, Wang J-J. 2021. Microbial community structure of waste water treatment plants in different seasons. Huan Jing Ke Xue 42:1488–1495. doi:10.13227/j.hjkx.202007015 33742946

[25] Serletti L, Dutcher L, Degnan KO, Szymczak JE, Cluzet V, David MZ, Cressman L, Glassman LW, Hamilton KW, Disease C, Prevention Prevention Epicenters P. 2023. Analysis of seasonal variation of antibiotic prescribing for respiratory tract diagnoses in primary care practices. Antimicrob Steward Healthc Epidemiol 3:e147. doi:10.1017/ash.2023.418 PMC10523546 37771744

[26] Gonzalez-Martinez A, Rodriguez-Sanchez A, Muñoz-Palazon B, Garcia-Ruiz M-J, Osorio F, van Loosdrecht MCM, Gonzalez-Lopez J. 2015. Microbial community analysis of a full-scale Demon bioreactor. Bioprocess Biosyst Eng 38:499–508. doi:10.1007/s00449-014-1289-z 25245398

[27] Velazquez-Meza ME, Galarde-López M, Cornejo-Juárez P, Bobadilla-Del-Valle M, Godoy-Lozano E, Aguilar-Vera E, Carrillo-Quiroz BA, Ponce de León-Garduño A, Velazquez Acosta C, Alpuche-Aranda CM. 2025. Bacterial communities and resistance and virulence genes in hospital and community wastewater: metagenomic analysis. Int J Mol Sci 26:2051. doi:10.3390/ijms26052051 PMC11900532 40076673

[28] Cai L, Ju F, Zhang T. 2014. Tracking human sewage microbiome in a municipal wastewater treatment plant. Appl Microbiol Biotechnol 98:3317–3326. doi:10.1007/s00253-013-5402-z 24305737

[29] Hendriksen RS, Munk P, Njage P, van Bunnik B, McNally L, Lukjancenko O, Röder T, Nieuwenhuijse D, Pedersen SK, Kjeldgaard J, et al.. 2019. Global monitoring of antimicrobial resistance based on metagenomics analyses of urban sewage. Nat Commun 10:1124. doi:10.1038/s41467-019-08853-3 PMC6408512 30850636

[30] Dekaboruah E, Suryavanshi MV, Chettri D, Verma AK. 2020. Human microbiome: an academic update on human body site specific surveillance and its possible role. Arch Microbiol 202:2147–2167. doi:10.1007/s00203-020-01931-x PMC7284171 32524177

[31] Jiao Y-N, Zhou Z-C, Chen T, Wei Y-Y, Zheng J, Gao R-X, Chen H. 2018. Biomarkers of antibiotic resistance genes during seasonal changes in wastewater treatment systems. Environ Pollut 234:79–87. doi:10.1016/j.envpol.2017.11.048 29169020

[32] Lee CR, Lee JH, Park M, Park KS, Bae IK, Kim YB, Cha CJ, Jeong BC, Lee SH. 2017. Biology of *Acinetobacter baumannii*: pathogenesis, antibiotic resistance mechanisms, and prospective treatment options. Front Cell Infect Microbiol 7:55. doi:10.3389/fcimb.2017.00055 PMC5346588 28348979

[33] Gómez P, Lozano C, Benito D, Estepa V, Tenorio C, Zarazaga M, Torres C. 2016. Characterization of staphylococci in urban wastewater treatment plants in Spain, with detection of methicillin resistant *Staphylococcus aureus* ST398. Environ Pollut 212:71–76. doi:10.1016/j.envpol.2016.01.038 26840519

[34] Ben Said M, Abbassi MS, Gómez P, Ruiz-Ripa L, Sghaier S, Ibrahim C, Torres C, Hassen A. 2017. *Staphylococcus aureus* isolated from wastewater treatment plants in Tunisia: occurrence of human and animal associated lineages. J Water Health 15:638–643. doi:10.2166/wh.2017.258 28771160

[35] Bengtsson-Palme J, Hammarén R, Pal C, Östman M, Björlenius B, Flach CF, Fick J, Kristiansson E, Tysklind M, Larsson DGJ. 2016. Elucidating selection processes for antibiotic resistance in sewage treatment plants using metagenomics. Sci Total Environ 572:697–712. doi:10.1016/j.scitotenv.2016.06.228 27542633

[36] Yang Y, Li B, Ju F, Zhang T. 2013. Exploring variation of antibiotic resistance genes in activated sludge over a four-year period through a metagenomic approach. Environ Sci Technol 47:10197–10205. doi:10.1021/es4017365 23919449

[37] Karkman A, Johnson TA, Lyra C, Stedtfeld RD, Tamminen M, Tiedje JM, Virta M. 2016. High-throughput quantification of antibiotic resistance genes from an urban wastewater treatment plant. FEMS Microbiol Ecol 92:fiw014. doi:10.1093/femsec/fiw014 26832203

[38] Wen Q, Yang L, Duan R, Chen Z. 2016. Monitoring and evaluation of antibiotic resistance genes in four municipal wastewater treatment plants in Harbin, Northeast China. Environ Pollut 212:34–40. doi:10.1016/j.envpol.2016.01.043 26840514

[39] Pärnänen KMM, Narciso-da-Rocha C, Kneis D, Berendonk TU, Cacace D, Do TT, Elpers C, Fatta-Kassinos D, Henriques I, Jaeger T, et al.. 2019. Antibiotic resistance in European wastewater treatment plants mirrors the pattern of clinical antibiotic resistance prevalence. Sci Adv 5:eaau9124. doi:10.1126/sciadv.aau9124 PMC6436925 30944853

[40] Rumky J, Kruglova A, Repo E. 2022. Fate of antibiotic resistance genes (ARGs) in wastewater treatment plant: preliminary study on identification before and after ultrasonication. Environ Res 215:114281. doi:10.1016/j.envres.2022.114281 36096165

[41] Liu Z, Klümper U, Liu Y, Yang Y, Wei Q, Lin JG, Gu JD, Li M. 2019. Metagenomic and metatranscriptomic analyses reveal activity and hosts of antibiotic resistance genes in activated sludge. Environ Int 129:208–220. doi:10.1016/j.envint.2019.05.036 31129497

