# Supplementary material for: Identification of antibiotic resistance genes and their bacterial hosts in samples collected from wastewater treatment plants in Palestine
Source: Microbiol Spectr. 2026 Sep 18;14(11):e00028-26. doi: 10.1128/spectrum.00028-26 (PMC13729141; doi:10.1128/spectrum.00028-26)
Supplement: Supplementary file 1 [file reviewer-comments.pdf]

ASM | Microbiology Spectrum

Metagenomic Identification of Antibiotic Resistance Genes and Bacterial Hosts in Wastewater Treatment Plants in Palestine

Mosab Hroub, Suheir Ereqat, Amer Al-Jawabreh, and Ziad Abdeen

Corresponding Author(s): Amer Al-Jawabreh, Arab American University

Review Timeline:

Submission Date:	February 11, 2026
Editorial Decision:	March 20, 2026
Revision Received:	May 28, 2026
Accepted:	July 20, 2026

Editor: Michael Becker

Reviewer(s): Disclosure of reviewer identity is with reference to reviewer comments included in decision letter(s). The following individuals involved in review of your submission have agreed to reveal their identity: suleiman fawaz al obeid (Reviewer #1)

Transaction Report:

(Note: With the exception of the correction of typographical or spelling errors that could be a source of ambiguity, letters and reports are not edited. The original formatting of letters and referee reports may not be reflected in this compilation.)

DOI: https://doi.org/10.1128/spectrum.00028-26

Re: Spectrum00028-26 (Metagenomic Identification of Antibiotic Resistance Genes and Bacterial Hosts in Wastewater Treatment Plants in Palestine)

Dear Prof. Amer Al-Jawabreh:

Thank you for the privilege of reviewing your work. Below you will find my comments, instructions from the Spectrum editorial office, and the reviewer comments.

I have now received the reviews of your manuscript and, while we appreciate the importance of the topic and the relevance of your study, the reviewers have identified several issues that should be addressed before the manuscript can be considered further for publication in Microbiology Spectrum.

Although one reviewer was broadly positive, the other raised several significant concerns, which I tend to agree with. These include issues related to methodological clarity and data analysis, particularly the lack of data normalization to aid interpretation, limited description of the bioinformatics pipeline, and absence of appropriate statistical analyses. Improvements to the writing and figure presentation would further improve the readability of the manuscript.

Please return the manuscript within 60 days; if you cannot complete the modifications within this time period, please contact me. If you do not wish to modify the manuscript and prefer to submit it to another journal, notify me immediately so that the manuscript may be formally withdrawn from consideration by Spectrum.

Revision Guidelines
To submit your modified manuscript, log into the submission site at https://spectrum.msubmit.net/cgi-bin/main.plex. Go to Author Tasks and click the appropriate manuscript title to begin. The information you entered when you first submitted the paper will be displayed; update this as necessary. Note the following requirements:

• Upload point-by-point responses to the issues raised by the reviewers in a file named "Response to Reviewers," NOT in your cover letter.
• Upload a compare copy of the manuscript (without figures) as a "Marked-Up Manuscript" file.
• Upload a clean .DOC/.DOCX version of the revised manuscript and remove the previous version.
• Each figure must be uploaded as a separate, editable, high-resolution file (TIFF or EPS preferred), and any multipanel figures must be assembled into one file.
• Any supplemental material intended for posting by ASM should be uploaded with their legends separate from the main manuscript. You can combine all supplemental material into one file (preferred) or split it into a maximum of 10 files with all associated legends included.

For complete guidelines on revision requirements, see our Submission and Review Process webpage. Submission of a paper that does not conform to guidelines may delay acceptance of your manuscript.

Data availability: ASM policy requires that data be available to the public upon online posting of the article, so please verify all links to sequence records, if present, and make sure that each number retrieves the full record of the data. If a new accession number is not linked or a link is broken, provide Spectrum production staff with the correct URL for the record. If the accession numbers for new data are not publicly accessible before the expected online posting of the article, publication may be delayed; please contact production staff (Spectrum@asmusa.org) immediately with the expected release date.

Publication Fees: For information on publication fees and which article types are subject to charges, visit ourwebsite. If your manuscript is accepted for publication and any fees apply, you will be contacted separately about payment during the production process; please follow the instructions in that e-mail. Arrangements for payment must be made before your article is published.

ASM Membership: Corresponding authors may join or renew ASM membership to obtain discounts on publication fees. Need to upgrade your membership level? Please contact Customer Service at Service@asmusa.org.

The ASM Journals program strives for constant improvement in our submission and publication process. Please tell us how we can improve your experience by taking this quick Author Survey.

Thank you for submitting your paper to Spectrum.

Sincerely,
Michael Becker
Editor
Microbiology Spectrum

Reviewer #1 (Comments for the Author):

Dear authors thank you for this very important study concerning the high resistance and spread in all the world please the abbreviation WWTPs should be indicated in the title or in the abstract.
The Antibiotic Resistance Genes and Bacterial Hosts in Wastewater Treatment Plants WWTPs in Palestine and other areas of the world are a very important threat to public health.

Reviewer #2 (Comments for the Author):

This manuscript presents a relevant and timely study using metagenomic sequencing to evaluate antibiotic resistance genes (ARGs) and resistant bacteria (ARB) in wastewater treatment plants (WWTPs) in Palestine. The study compares seasonal variations and evaluates the efficiency of ARG/ARB removal in two facilities using activated sludge systems. I commend the authors for carrying out this work in such difficult times for their country and region. My solidarity with them.

While the research question is important and the methodology is generally appropriate, the manuscript requires substantial revision, particularly in scientific writing, methodological clarity, data normalization, and statistical analysis.

Major points for revision:

- Language and grammar: The manuscript contains frequent grammatical and form errors that limit clarity. A comprehensive language review by a fluent English speaker or a professional editing service is strongly recommended.

- Lack of normalization for sequencing depth: The study reports ARG and ARB abundance as absolute read counts across samples. However, sequencing depth varies by more than 100-fold across samples (e.g., winter vs. summer influents), significantly biasing the interpretation of gene and species abundances. The authors should normalize ARG and ARB abundances to total sequencing reads (e.g., per million reads or relative proportions) to allow for meaningful comparisons across conditions.

- Bioinformatics pipeline - Methodological transparency: The authors briefly describe the workflow but omit key details, such as quality control and filtering steps, software versions and parameters, and the method for associating ARGs with bacterial hosts, particularly given the confounding potential of horizontal gene transfer.

- Statistical analysis missing: Although SPSS is mentioned, no statistical tests are reported. The authors should perform appropriate statistical tests (e.g., t-tests, nonparametric tests, or ANOVA) to support comparisons across sites and seasons.

- Overlap between results and discussion: The discussion reiterates many results without providing sufficient critical interpretation. The authors should focus more on: (1) Biological significance and risk implications of observed ARGs, (2) comparative insights from other regional or global WWTP studies, (3) recommendations for improving monitoring and policy.

Minor Suggestions:

- Clarify the treatment differences between the two WWTPs (activated sludge vs. aeration lagoon).

- Ensure all ARG names are consistently italicized and formatted.
Improve figure captions for Figures 1-4 and ensure axis labels are legible and units are defined.

Conclusion:

This manuscript presents meaningful data from an underrepresented region and addresses the global issue of environmental antibiotic resistance in WWTPs. However, several methodological and interpretive shortcomings (especially the lack of data normalization) must be addressed before the manuscript can be considered for publication.

Metagenomic Identification of Antibiotic Resistance Genes and Bacterial Hosts in Wastewater Treatment Plants in Palestine

Mosab Hroub[1], Suheir Ereqat[2], Amer Al-Jawabreh[3,4*], and Ziad Abdeen[5]

1 Hebron University, Al-Khalil, Palestine

2 Biochemistry and Molecular Biology Department, faculty of Medicine, Al-Quds University, Jerusalem, Palestine

3 Leishmaniases Research Unit-Jericho, Palestine

4. Arab American University, Palestine

5. Al-Quds Public Health Society, Palestine

***** Corresponding author: Al-Jawabreh A.; islahjr@yahoo.com, amer.aljawabreh@aaup.edu; Jericho, Palestine

ABSTRACT

Wastewater treatment plants (WWTPs) are recognized as significant sources for the dissemination of antibiotic-resistant bacteria (ARB) and antibiotic resistance genes (ARGs). In the West Bank, Palestine, four WWTPs are currently working in Jenin, Tulkarem, Ramallah (Al-Terah), and Al-Bireh, most of which use secondary treatment processes based on activated sludge. In this study, Illumina high-throughput sequencing was used to characterize the profiles of ARB and ARGs in the Al-Bireh and Al-Terah WWTPs. Raw wastewater (influent) and secondary-treated water (effluent) samples were collected during two seasons, summer and winter. The results revealed a higher abundance of ARB and greater diversity of ARGs in winter compared to summer. The most dominant species detected were *Acinetobacter baumannii* in Al-Bireh samples and *Pasteurella multocida* in Al-Terah samples. In total, 138 distinct ARGs providing resistance to 12 antibiotic classes were identified, with genes providing resistance to macrolides and tetracyclines were the most prevalent. The removal efficiency of the ten most common ARB and ARGs ranged from 85% to 100% in both treatment plants. In conclusion, unfiltered antibiotics and ARGs in treated wastewater can be absorbed by plants and crops, posing a potential public health risk. The abundance

of ARGs and their bacterial hosts was higher in winter, highlighting the need to enhance sanitation measures and improve treatment efficiency. Strengthening wastewater treatment systems should be a priority for policymakers to prevent the spread of ARB and ARGs in Palestine.

IMPORTANCE Untreated wastewater plays a key role in spreading antibiotic-resistant bacteria (ARB) containing antibiotic-resistant genes (ARG) in the environment. The current study characterized the ARB and ARG in two wastewater treatment plants (WWTP) in Jenin and Ramallah, Palestine in summer and winter in both pre-treated and post-treated water. In winter, ARB were more abundant, and ARG were highly diverse in both plants alike. *Acinetobacter baumanii*, an extremely drug-resistant bacterium, was found as a dominant bacteria. The study revealed 12 ARG groups based on the antibiotic class in the two WWTP, with β-lactamases showing the highest number of resistant genes. The efficiency of removal of bacteria in both plants ranged from 85-100%.

KEYWORDS: ARGs, ARB, MDR, wastewater treatment, activated sludge, influent, effluent

INTRODUCTION

Wastewater treatment (WWT) is a process designed to remove pollutants and contaminants from wastewater (WW) through biological, chemical, and physical methods. Once treated, the wastewater can be safely discharged into the environment. Sludge, a by-product of the treatment process derived from sewage, requires further processing before reuse or disposal. It consists of two types: sludge and activated sludge, both containing various organic and inorganic compounds. Sludge has a high water content originating from liquid wastewater with a small proportion of solid waste. It can be a primary sludge that produced during the primary treatment stage, or secondary sludge, resulting from the secondary treatment process. Activated sludge (AS) contains different types of microorganisms that live in harmony together. The microbial diversity in AS is higher than that in incoming wastewater (IW) and includes many uncultivable species. The main difference between sewage and

sludge is that sewage is a suspension of solid and liquid waste, whereas sludge refers to the solid material separated from that suspension (1).

The WW treatment process consists of three main stages: the physicochemical process as the primary step, the biological process as the secondary step, and a tertiary process that involves advanced methods such as the advanced oxidation process. The main objective of these steps is to produce high-quality water suitable for reuse in various applications (2).

A huge diversity of pathogenic and nonpathogenic microorganisms is found living together in wastewater, sharing metabolic activities to support the growth and multiplication of one another. The WWTPs became a hotspot for the presence of microorganisms, especially the antibiotic-resistant organisms that come from humans /animals and are released into sewage through feces, urine, dead bodies, and manure. Moreover, the WW that flows from hospitals and farming facilities could be the major source of antibiotic resistance genes (ARGs) and antibiotic-resistant bacteria (ARB) that are released in the environment (3).

The main route of transfer of resistance in the AS occurs by horizontal transfer of ARGs through mobile genetic elements (MGEs) such as plasmids, transposons, bacteriophages, insertion sequences, and integrons(4). There are many ARGs that encode for resistance against different antibiotics like tetracyclines (tet), sulfonamides (sul), β-lactams (bla), macrolides (erm), aminoglycosides (aac), fluoroquinolone (fca), colistin (mcr), vancomycin (van), and multidrug (mdr) (5). It was shown that MGEs were abundant in the sludge samples, and this may be important for the acquisition and mobility of various ARGs among the bacterial species (6). The spreading of ARGs and ARB in the environment could lead to antibiotic resistance in humans and threaten public health (3).

In the past, culture-based identification methods faced challenges in analyzing microbial communities from environmental samples, as they relied mainly on morphological characteristics for microorganism identification. Additional limitations of these methods include the low selectivity of

culture media and the need for diverse culture conditions, which often inhibit or prevent the growth of approximately 99% of the bacterial community present in wastewater (7, 8).

On the other hand, molecular methods are benefiting in the determination of microorganisms and their biological activity under different situations. They allow the real-time observation of the microorganisms and their metabolic pathways. Accordingly, the shotgun metagenomic method is capable of determining the whole genomic DNA in environmental samples without the need to isolate or culture individual microorganisms in the laboratory(9). This contrasts with the classical PCR method, which depends on specific primers to isolate ARGs and detect their abundance in the bacteria. Moreover, classical PCR is limited in detecting novel ARGs compared to next-generation metagenomic analysis (10). Furthermore, the metagenomic approach can detect the low-abundance microorganisms by generating hundreds to thousands of sequences and give a clear picture of the microbial community found in the sample (11). Using metagenomic analysis, this study aimed to profile ARB and their associated antibiotic ARGs isolated from two WWTPs across two seasons, analyzing both pre- and post-treatment samples, and to evaluate the removal efficiency of the selected WWTPs in Palestine.

MATERIALS AND METHODS

WWTPs description

The water samples were collected from both Al-Bireh and Al-Terah WWTPs, which are located in Ramallah city. Al-Bireh plant was constructed in 1997 and started functioning in February 2000. It has a capacity of 6000 m^3/day of treated wastewater, and serves about 50000 people in Al-Bireh town. This WWTP depends on the conventional activated sludge method as a secondary treatment process, including a low-loaded activated sludge stage, which, besides the removal of carbonaceous compounds, it performs oxidation of nitrogen compounds (nitrification). The treatment process is performed in a unit composed of a mixing and distribution chamber for aeration tank feeding without the involvement of tertiary treatment process. However, the effluent output of this plant is released

into the wadies. Al-Terah plant located in Al-Terah town and serves 47.500 population. The capacity of this plant is 1370 m^3/day. It depend on two aeration tanks to treat wastewater. However, the treated water is deposited in Wadi Bitunia (12).

Sampling

Two different types of samples from both WWTPs were collected using sterile 500ml bottles (Duran, Inc. USA). From each WWTP, one raw (influent) and one treated (effluent) sample were collected in two different seasons: winter (February) and summer (August). The composite sampling involved taking equal subsamples every five meters in sterile bottles to finally have the whole sample to ensure representativeness, then transported within 48 hours in a cool box to the laboratory for DNA extraction.

Samples preparation

Influent samples (500ml) were first divided equally into ten samples using 50ml sterile Falcon tubes, then centrifuged at maximum speed, about 3220 g, for 20 minutes in a 5810 R centrifuge (Eppendorf, Germany) to obtain the pellet. The pellets were reconstituted with absolute ethanol in a ratio of 1:2 and kept in -20°C to be used for DNA extraction (Ma, Li et al. 2017).

The 500ml effluent samples were filtered using a Polyvinylidene difluoride (PVDF) membrane filter (Stericup 250 ml, Durapore, 0.45 μm PVDF). The membrane then then removed carefully using sterile scissors, and forceps, transferred to sterile tubes, and kept at -20°C to be used for DNA extraction.

DNA extraction and concentration

Influent samples

The 50ml frozen samples were thawed, homogenized separately by vortex mixer, and finally the ten tubes were collected in one tube. For DNA extraction, QIAamp® DNA Mini Kit (Qiagen GmbH, Germany) was used according to manufacturer’s instructions of bacterial DNA extraction protocols,

except in the final step, in which 50µl of elution buffer was added instead of 200µl to obtain a final extract volume of 50µl.

Effluent samples

The filter papers were thawed, carefully removed from the tube, and cut into small pieces using sterile scissors. The DNA was extracted from filter papers using the same kit and the same procedures used for influent samples. The concentration of DNA samples was measured using the Qubit v4 (Invitrogen, USA) machine. DNA concentration was adjusted to 0.4 ng/µl to be accepted for library preparation.

Library preparation

For metagenomic analysis, the DNA library was prepared using the Nextera XT DNA library preparation kit (illumina®, USA). Briefly, the gDNA was normalized to have five microliters of DNA. Tagmentation was done using five µl of Tagmentation buffer (Amplicon Tagment Mix) with a special Tagmentation mixture that contains transposomes and then incubated at 55℃ for five minutes. Five µl of Neutralize Tagment Buffer (NT) was added to the mix and incubated for five minutes at room temperature. Two different index adapters were added (five microliters each) at both ends of the DNA fragment. To attach those index adapters to the DNA, a 15µl of Nextra PCR master mix were added to 35µl of tagmented DNA to a final volume of 50µl, the reaction mixture was subjected to thermal cycling as follows: 72℃ for 3 minutes, 95℃ for 30 seconds, then 12 cycles of 95℃ for 10 seconds, 55℃ for 30 seconds, 72℃ for 30 seconds, then 72℃ for 5 minutes, and finally put on hold at 10℃. The obtained DNA library with fragments between 300-500bp was then purified with 1X AMPure XP beads (Beckman Coulter, Brea, CA, USA). The concentration of the prepared DNA library was measured using the Qubit v4 (Invitrogen, USA) machine. The quality of the library was evaluated by the Tapestation machine (Agilent, CA, USA), and the library was normalized to

four nmol. Sequencing (as a single read) was done using the NextSeq 500/550 High Output Kit v2.5 (150 Cycles).

Bioinformatics analysis

Analysis workflow

The obtained fastaq files were converted into Fasta format using the FASTQ to FASTA converter from the FASTX- toolkit command on the online Galaxy software (www.usegalaxy.org).. The annotation process was done using the ABRicate command, which performs mass screening of contigs for antimicrobial and virulence genes. The output results of this command included the related reference sequence which was indicated by the GenBank accession number. The accession number accompanied with each ARG from the previous Galaxy workflow analysis was BLAST searched (www.blast.ncbi.nlm.nih.gov) to identify the bacterial host. Each organism with an identity and query cover above 97% was included (13). After downloading the results file of the ABRicate, the statistical package SPSS v29 (IBM Corporation, USA) was used to measure the frequency of each ARGs. Then 'Cross Tab' command was applied to link the accession number to different types of ARGs (Appendix 2).

ARGs analysis

AMR gene family, drug class, and resistance mechanisms for each gene were analyzed using 'The Comprehensive Antibiotic Resistance Database (CARD, https://card.mcmaster.ca/), The 'Analyze' command on the CARD main page was chosen, then the reference gene identifier (RGI) was selected to search for resistant genes using the GenBank accession numbers obtained from the previous Galaxy workflow analysis.

Removal efficiency

The removal efficiency of ARGs was calculated by the following equation described elsewhere (14).

$$\%\text{Removal Efficiency} = \frac{\text{Influent frquency}}{\text{Influent freq} - \text{Effluent freq}} \text{x}100\%$$

RESULTS

Our study utilized a deep metagenomic sequencing approach to analyze the ARGs present in influent and effluent samples collected from both the Al-Bireh and Al-Terah wastewater treatment plants across two different seasons. During winter, the total number of sequencing reads was higher in Al-Terah WWTP than in Al-Bireh for both influent and effluent samples. Conversely, in summer, Al-Bireh exhibited a greater number of reads than Al-Terah in both sample types. (Table 1).

Table 1. The overall metagenomics characteristics in the two WWTPs, Al-Bireh and Al-Terah, in winter and summer

	Al-Bireh WWTP		Al-Terah WWTP	
	Winter	Summer	Winter	Summer
No. reads, influent	$3.7\text{x}10^6$	$0.37\text{x}10^6$	$5.09\text{x}10^6$	$0.031\text{x}10^6$
No. reads, effluent	$0.095\text{x}10^6$	$0.26\text{x}10^6$	$0.32\text{x}10^6$	$0.022\text{x}10^6$
No. ARGs, influent	400	253	305	162
Prevalent ten, %	44	38	51	56
ARGs	(msr(E), mph(E), tet(Q), cfxA2, tet(39), sul1, erm(F), mph(A), tet(Q), aph(6)-Id)	(msr(E), mph(E), mph(A), sul1, aph(6)-Id, tet(39), cfxA2, ant(3")-Ia, cmlA1, nd msr(D)	(msr(E), mph(E), sul1, tet(39), tet(Q), mph(A), qnrS2, aph(6)-Id, cmlA1, and cfxA2	(msr(E), mph(E), tet(39), tet(Q), sul1, sul2, mef(C), cfxA2, aph(3")-Ib, and mef(A)
Antibiotics, influent	Macrolide, tetracycline, Beta-lactamase, sulfonamide, streptogramin , aminoglycoside	Macrolide, tetracycline, aminoglycoside, Sulfonamide Trimethoprim	Macrolide, Sulfonamide ,tetracycline, aminoglycoside, Chloramphenicol/florfenicol, Beta-lactamase Quinolone	Macrolide, Sulfonamide ,tetracycline, aminoglycoside, Beta-lactamase
No. ARGs, effluent	88	72	97	52

Prevalent ten, %	67	51	62	66
ARG subtypes	msr(E), mph(E), tet(39), sul1, sul2, erm(F), ant(3")-Ia, oqxB_, mef(C), and mph(G)	msr(E), mph(E), mph(A), sul1, ere(A), tet(C), cfxA2, ant(3")-Ia, blaOXA-10, blaLCR-1	msr(E), oqxB, mph(E), sul1, ere(D), sul2, tet(39), erm(F), ant(3")-Ia, and floR	msr(E), mph(E), sul1, ant(3")-Ia,erm(F),qepA1, aadA10, aadA2,dfrA1, erm(35)
Antibiotics, effluent	Macrolide, Tetracycline, Sulfonamide, streptogramin , aminoglycoside	Macrolide, Beta-lactamase, Sulfonamide tetracycline, aminoglycoside	Macrolide,Tetracyline, aminoglycoside, Chloramphenicol/florfenicol, sulfomamide	Macrolide,sulfonamide aminoglycoside,

ARGs abundance

In winter, 400 antibiotic resistance gene (ARG) subtypes were identified in the influent samples from the Al-Bireh WWTP. The ten most prevalent ARGs accounted for 44% of the total and, in order of high abundance, included msr(E), mph(E), tet(Q), cfxA2, tet(39), sul1, erm(F), mph(A), tet(Q), and aph(6)-Id (Fig. 1, Table 1). These ARGs conferred resistance to several antimicrobial drug families, predominantly macrolides and tetracyclines, followed by β-lactams, sulfonamides, streptogramins, and aminoglycosides. In the influent samples from the Al-Terah WWTP, 305 ARG subtypes were detected, with the ten most prevalent representing 51% of the total. These included msr(E), mph(E), sul1, tet(39), tet(Q), mph(A), qnrS2, aph(6)-Id, cmlA1, and cfxA2 (Fig. 1, Table 1). The ARGs in Al-Terah conferred resistance to antibiotic families similar to those in Al-Bireh, with some differences such as the presence of quinolone resistance genes, which were detected only in Al-Terah.

During summer, 253 ARG subtypes were identified in influent samples from Al-Bireh WWTP, with the ten most dominant ARGs comprising 38% of the total. These included msr(E), mph(E), mph(A), sul1, aph(6)-Id, tet(39), cfxA2, ant(3")-Ia, cmlA1, and msr(D). Similar to winter, macrolide resistance genes were the most abundant, followed by those conferring resistance to tetracyclines,

aminoglycosides, sulfonamides, and trimethoprim. In Al-Terah WWTP during summer, 162 ARG subtypes were detected, with the ten most prevalent accounting for 56% of the total. These were msr(E), mph(E), tet(39), tet(Q), sul1, sul2, mef(C), cfxA2, aph(3")-Ib, and mef(A). The resistance gene families identified were consistent with those found in the Al-Bireh samples.

Figure 1: Difference in abundance (No. of reads) of ARGs between winter and summer found in influent samples taken from Al-Bireh and Al-Terah WWTPs

In winter, the effluent samples from the Al-Bireh WWTP contained 88 ARG subtypes, with msr(E), mph(E), tet(39), sul1, sul2, erm(F), ant(3")-Ia, oqxB, mef(C), and mph(G) comprising 67% of the total ARGs (Fig. 2, Table 1). The most abundant ARG families were consistent with those detected in the influent samples. In contrast, the Al-Terah WWTP effluent contained 97 ARG subtypes, with msr(E), oqxB, mph(E), sul1, ere(D), sul2, tet(39), erm(F), ant(3")-Ia, and floR representing 62% of the total. These ARGs conferred resistance to antibiotic families similar to those found in the influent samples.

In summer, the effluent samples collected from the Al-Bireh and Al-Terah WWTPs revealed 72 and 52 ARG subtypes, respectively. The ten most prevalent ARGs accounted for 51% of the total in Al-Bireh and 66% in Al-Terah (Fig. 2, Table 1). The dominant ARGs in both plants were associated with resistance to macrolide, sulfonamide, tetracycline, and aminoglycoside antibiotic classes (Appendix 2).

Figure 2: Differences in abundance (No. reads) of ARGs in winter compared to summer in effluent samples from Al-Bireh and Al-Terah WWTPs.

Identification of ARGs bacterial hosts and removal efficiency

In winter, 53 and 100 different bacterial species were found in influent samples from Al-Bireh and Al-Terah WWTPs, respectively. Ten bacterial species were found to comprise 74% and 77% of the total species abundance in Al-Bireh and Al-Terah plants, respectively. Regarding phyla frequency, the most abundant phyla were *Pseudomonadota* (67.8%), *Firmicutes* (8.4%), and *Bacteroidota* (6.3%). Uncultivable bacteria represented 11.3 and 11% in Al-Bireh and Al-Terah WWTPs, respectively. According to bacterial genera and species, *Acinetobacter baumannii* had the highest frequency in Al-Bireh WWTP compared to *Pasteurella multocida* in Al-Terah WWTP. While in summer, the influent

Figure 3: Differences in abundance (No. of reads) of major ARB species between winter and summer in influent samples taken from Al-Bireh and Al-Terah WWTPs.

samples from Al-Bierh WWTP contained 30 different bacterial species, with the most prevalent ten comprising 81% of the total bacterial species. However, in the Al-Terah influent sample, 68 species were found, with the most frequent ten comprising 76% of the total. At the phylum level, the most abundant phyla were *Pseudomonadota* (72%), *Firmicutes* (12-13%), uncultivable bacterium (10-11%), and *Bacteroidota* (2%) in the influent samples from Al-Bireh and Al-Terah samples.

On the other hand, in winter effluent sample, only eight bacterial species were found in Al-Bireh and 52 in Al-Terah WWTPs. The most common phyla were *Pseudomonadota* (63.55%) and *Bacteroidota* (6.3%), while uncultivable bacteria represented 1.27% in both Al-Bireh and Al-Terah plants, respectively. In the Al-Terah plant, *Firmicutes* (5%) and *Actinobacteria* (7%) were additionally found. However, in summer, the effluent sample from Al-Bireh WWTP contained only two bacterial species: *A. baumannii* and K. pneumoniae, belonging to the phylum *Pseudomonadota*. In contrast, the Al-Terah effluent sample was shown to contain 21 bacterial species. The dominant phyla were *Pseudomonadota*

(58%) followed by *Firmicutes* (13 %), *Bacteroidota* (3 %), and *Actinobacteria* (2%), whereas uncultivable bacterium comprised 22 % (Appendix 2).

Figure 4: Difference in abundance of the major bacterial species (No. of reads) between winter and summer in effluent samples from Al-Bireh and Al-Terah WWTPs.

In the samples from Al-Bireh WWTP, some of the bacteria such as *Prevotella* species and *Streptococcus pneumoniae*, were cleared from the effluent sample, and thus the removal efficiency was considered 100%. The removal efficiency for the other bacteria was still high ranging from 67-100%. While in Al-Terah WWTP samples, only *Acinetobacter johnsonii* has been cleared completely, with the removal efficiency ranging from 63 to100%. (Appendix 1). The removal efficiency of ARGs ranged from 85-100% in Al-Bireh WWTP and 87-100% in Al-Terah WWTP.

There are 12 ARG classification groups based on the antibiotic class. The most diverse one that comes with different resistance genes is beta-lactamase with 34 different genes, followed by tetracycline and aminoglycoside, which have 20 and 18 genes, respectively. The other group was ordered according to the number of genes. (Appendix-2).

DISCUSSION

As antibiotics were used for many years to eliminate bacteria, which wide range of these bacteria have acquired resistance to antibiotics by horizontal and vertical gene transfer (HGT) and mutations (15). This has introduced antibiotic ARB and ARG on a large scale into the environment, becoming an emerging global health problem (3, 16, 17). This gives the reason for estimating the level of ARGs in the activated sludge that ends up in the environment. The study aimed to document the presence of various types of ARB and ARGs in raw influent wastewater (WW) and treated effluent WW collected in two different seasons, winter and summer, from two WWTPs. The sources of raw wastewater that flow

to those plants are houses, slaughterhouses, and different hospitals in the area. In this study, a difference was observed in the amount of bacterial DNA, as represented by the number of bacterial reads, between influent and effluent samples across the two seasons. The decreased number of reads from thousands of reads prior to treatment to a couple of hundred post-treatment may reflect, to some extent, the efficiency of the treatment plant. However, the remaining amount of ARG in effluent can still spread in the environment with high risk potential (18). On the other hand, the reason for the high amount of bacterial DNA in winter rather than summer is due to rainwater that carries most of the microorganisms in the sewer pipeline and goes directly to plants. Moreover, the prevalence of bacterial infection is usually higher in winter than summer, especially among vulnerable people with weak immunity, such as children and the elderly, who may overuse broad-spectrum antibiotics and thus developing resistant bacteria (18, 19). Also, most bacteria, including ARB, that cause gastrointestinal, respiratory tract, and urinary tract infections are shed into the sewage system. In addition, the overuse of antibiotics results in excretion of unmetabolized antibiotics into the environment, which with the different pressures of pollutants, causes the spread of ARGs among bacterial communities in the environment (20). In the Al-Bireh plant, the treatment process depends basically on activated sludge which uses bacteria to degrade the contaminants and thus the microbial community. While in Al-Terah depend mainly on two aeration lagoons to treat water.

In winter, more bacterial species (54,100) were detected in the influent samples than in summer samples (3,068) taken from both Al-Bireh and Al-Terah WWTPs, respectively. Most of the detected in the influent and effluent samples were gram-negative bacteria, including Salmonella enterica and Pseudomonas aeruginosa (Fig 3 and 4), the WHO priority pathogens (21). The detected ARB are the most common causing disease and may survive at a wide range of temperatures and pH, such as Pseudomonas aeruginosa, with a growth range between 4.0 and 42°C and pH of 4.5 to 9.0 (22). He and

colleagues (23) showed that the amount and diversity of bacteria in winter were more than in summer, which is explained by increasing the biomass of the reactor to maintain the effectiveness of wastewater treatment under cold temperature, besides the increase of antibiotic use during winter, which may increase the number of resistant bacteria (24). At the phylum level, the most abundant ARB found in both seasons were *Pseudomonadota*, followed by *Firmicutes* and *Bacteroidota*. Of the *Pseudomonadota*, several significant species were identified, such as *Acinetobacter baumannii*, *Escherichia coli*, *Pasturella multocida*, and K. *pneumoniae*. However, these species were significantly more frequent in winter than in summer. Our results are consistent with several other studies (8, 25) and inconsistent with Ju et al study which reported that the most abundant phylum in the influent sample was *Firmicutes*, followed by *Pseudomonadota*, *Actinobacteria*, and *Bacteroidota* (26). In regards to genera frequencies, our results were in agreement with those reported by other studies (27), which showed that the most abundant bacterial genus across the samples was *Acinetobacter*, and disagreed with other results stating that the most frequent genera were Bacteroides, *Escherichia*, *Streptococcus* (27). Ahmed et al reported that the most dominant bacterial genera in raw wastewater samples were Pseudomonas, followed by Arcobacter and Bacteroides (8). In our study, all the bacterial species were found to match the pattern of human pathogens which cause different infections in the respiratory tract, gastrointestinal, and urinary tracts (28).

The effluent samples contained eight and 52 species in the winter sample compared to three and 21 species in the summer samples from Al-Bireh and Al-Terah, respectively, which is runs parallel to the distribution of ARGs. Most of them belonged to the bacterial phyla Pseudomonadota, followed by Bacteroidota, with lower relative abundance than the influent samples. Our results, inconsistent with another study (29) showed that the most abundant phylum Pseudomonadota was less frequent in winter than in summer. In the Al-bireh sample, *Acinetobacter baumanaii*, a commonly known multidrug-

resistant (MDR) *Proteobacteria* responsible for hospital-acquired infection (HAI), had the highest frequency in both seasons, while other studies showed that *Pseudomonas aeruginosa*, another MDR-HAI bacteria, was the most frequent. (8, 27, 30). In contrast, in Al-Terah samples, *Pasteurella multocida*, an animal-related opportunistic commensal *Pseudomonadota*, was found at high frequency. There were also some bacteria explored that pose a significant health impact, but not in the top 10 bacteria. Staphylococcus aureus (S. aureus) is considered to be an opportunistic pathogen that causes infection. Methicillin-resistant *Staphylococcus aureus* (MRSA) is the most significant type because it harbors different ARGs. S. aureus harbors different ARGs like msr(A), Inu(B), and tet(M), similar to our finding (31, 32).

In this study, we noticed that most of the bacteria were efficiently removed after treatment, which could be attributed to the high performance of plants, especially in the Al-Bireh than Al-Terah plant. Al-Bierh WWTPs it is based on using an aeration tank and activated sludge as a secondary process under the effect of oxygen, and the other uses an aeration lagoon, which depends on the oxygen pumped and circulated to build aerobic conditions to treat water. Moreover, it relies on a sedimentation process in which the pathogens are adsorbed in the biosolid phase (8). A dramatic decrease in the anaerobic bacteria was noted after treatment, which may be due to shifting from an anaerobic to aerobic conditions, replacing the anaerobic bacteria with facultative anaerobic ones with the aid of low temperature, and thus bacteria that live under oxygen condition will proliferate and be retained in the plant (33).

In the influent samples collected from Al-Bireh and Al-Terah, 400,305 ARGs subtypes were found in the winter samples compared to 253,162 ARGs subtypes found in summer. On the other hand, the effluent samples from the two plants, Al-Bireh and Al-Terah, contained effectively less ARGs than in the influent samples, with higher abundance of ARGs in summer (88 and 97, respectively) than in winter

(77 and 52, respectively) (Table 1, Appendix 2). The 1.6-fold ARG increase in winter could be explained by intensive use of antibiotics (34). However, a few studies showed that temperature differences between seasons have very little or no impact on ARG abundance (35, 36). Our study showed that the most abundant resistant genes in the two were msr(E) and mph(E), and to a lesser degree tet(Q). These genes were reported as macrolide resistance genes, especially to erythromycin antibiotics, including azithromycin, clarithromycin, and spiramycin (Fig. 3 and 4) which is in congruence with other studies (27, 34, 37). Several studies revealed different patterns of predominating resistant genes such as tet_Q, tetA, tetX (tetracycline), ermB (erythromycin), sul1 (sulfonamide), blaCTX-M (cefotaxime), and mecA (methicillin) (8, 38). The discrepancy in ARG profiles between studies can be explained by the different wastewater sources and treatment processes of WWTPs, the class of antibiotics consumed by the served community, and the completeness of ARG databases that rely on the depth of metagenomic sequencing (39).

Conclusion

Shotgun metagenomic analysis offers a clear advantage over classical PCR methods by enabling the detection of uncultivable bacteria and the identification of novel ARGs without targeting specific bacterial sequences. In this study, several pathogenic bacterial species were identified, including *Acinetobacter baumannii*. The abundance of ARGs and their associated bacterial hosts was higher in February compared to August. Although the overall removal efficiency in the studied wastewater treatment plants was high, the persistence and dissemination of unfiltered ARB and ARGs into the environment remain a major concern. These residual contaminants can infiltrate groundwater, surface water, soil, and crops, posing a significant threat to public health. Therefore, it is crucial to raise public awareness about the risks associated with wastewater containing high levels of pathogens and antibiotic resistance determinants. In addition, enhancing sanitation infrastructure and improving the efficiency of

wastewater treatment systems should be a priority for policymakers to mitigate the spread of ARB and ARGs in treated wastewater in Palestine.

ACKNOWLEDGMENT

This work was partially supported by the U.S Department of State under the identifier SIS70022GR0033 grant - Pharmaceuticals M35-018.

DATA AVAILABILITY

All data are available as appendix files.

REFERENCES

1. Poblete IBS, Araujo OdQF, de Medeiros JL. 2022. Sewage-Water Treatment and Sewage-Sludge Management with Power Production as Bioenergy with Carbon Capture System: A Review. Processes 10:788.
2. Fernandes J, Ramísio PJ, Puga H. 2024. A Comprehensive Review on Various Phases of Wastewater Technologies: Trends and Future Perspectives. Eng 5:2633-2661.
3. Garcia-Torné M, Falcó I, Borrell X, Bautista A, Mazigh R, Aznar R, Sánchez G, Farré M, Llorca M. 2025. Comprehensive Study of Antibiotics and Antibiotic Resistance Genes in Wastewater and Impacted Mediterranean Water Environments. Antibiotics 14:341.
4. Yu Z, Liu Z, Sun L, Dong C, Jin Y, Hu B, Cheng D. 2024. Mobile genetic elements mediate the cross-media transmission of antibiotic resistance genes from pig farms and their risks. Science of The Total Environment 926:172115.
5. Jian Z, Zeng L, Xu T, Sun S, Yan S, Yang L, Huang Y, Jia J, Dou T. 2021. Antibiotic resistance genes in bacteria: Occurrence, spread, and control. Journal of Basic Microbiology 61:1049-1070.
6. Guo J, Li J, Chen H, Bond PL, Yuan Z. 2017. Metagenomic analysis reveals wastewater treatment plants as hotspots of antibiotic resistance genes and mobile genetic elements. Water Research 123:468-478.
7. Hladilek MD, Gaines KF, Novak JM, Collard DA, Johnson DB, Canam T. 2016. Microbial community structure of a freshwater system receiving wastewater effluent. Environ Monit Assess 188:626.
8. Ahmed W, Staley C, Sidhu J, Sadowsky M, Toze S. 2016. Amplicon-based profiling of bacteria in raw and secondary treated wastewater from treatment plants across Australia. Applied microbiology and biotechnology 101:1253-1266.
9. Chen Y, Fan LC, Chai YH, Xu JF. 2022. Advantages and challenges of metagenomic sequencing for the diagnosis of pulmonary infectious diseases. Clin Respir J 16:646-656.
10. Schmieder R, Edwards R. 2012. Schmieder R, Edwards R.. Insights into antibiotic resistance through metagenomic approaches. Fut Microbiol 7: 73-89. Future microbiology 7:73-89.
11. Ahn Y, Choi J. 2016. Bacterial Communities and Antibiotic Resistance Communities in a Full-Scale Hospital Wastewater Treatment Plant by High-Throughput Pyrosequencing. Water 8:580.
12. Y. Mogheir TAH, Z. Zomlot , A. Ahmed and D. Fatta. 2005. Treated Wastewater Reuse in Palestine.
13. Ravi A, Ereqat S, Al-Jawabreh A, Abdeen Z, Abu Shamma O, Hall H, Pallen MJ, Nasereddin A. 2019. Metagenomic profiling of ticks: Identification of novel rickettsial genomes and detection of tick-borne canine parvovirus. PLoS neglected tropical diseases 13:e0006805-e0006805.

14. Freeman CN, Scriver L, Neudorf KD, Truelstrup Hansen L, Jamieson RC, Yost CK. 2018. Antimicrobial resistance gene surveillance in the receiving waters of an upgraded wastewater treatment plant. FACETS 3:128-138.
15. Amarasiri M, Takezawa T, Malla B, Furukawa T, Sherchand JB, Haramoto E, Sei K. 2022. Prevalence of antibiotic resistance genes in drinking and environmental water sources of the Kathmandu Valley, Nepal. Front Microbiol 13:894014.
16. Ying GG, He LY, Ying AJ, Zhang QQ, Liu YS, Zhao JL. 2017. China Must Reduce Its Antibiotic Use. Environ Sci Technol 51:1072-1073.
17. Li P, Wu Y, He Y, Zhang B, Huang Y, Yuan Q, Chen Y. 2020. Occurrence and fate of antibiotic residues and antibiotic resistance genes in a reservoir with ecological purification facilities for drinking water sources. Sci Total Environ 707:135276.
18. Luo C, Zhang T, Mustafa MF, Li M, XU S. 2025. Removal efficiency of ARGs in different wastewater treatment plants and their potential risks in effluent. npj Clean Water 8.
19. Bonanno Ferraro G, Bonomo C, Brandtner D, Mancini P, Veneri C, Briancesco R, Coccia AM, Lucentini L, Suffredini E, Bongiorno D, Musso N, Stefani S, La Rosa G. 2024. Characterisation of microbial communities and quantification of antibiotic resistance genes in Italian wastewater treatment plants using 16S rRNA sequencing and digital PCR. Sci Total Environ 933:173217.
20. Sharma VK, Johnson N, Cizmas L, McDonald TJ, Kim H. 2016. A review of the influence of treatment strategies on antibiotic resistant bacteria and antibiotic resistance genes. Chemosphere 150:702-714.
21. Drane K, Sheehan M, Whelan A, Ariel E, Kinobe R. 2024. The Role of Wastewater Treatment Plants in Dissemination of Antibiotic Resistance: Source, Measurement, Removal and Risk Assessment. Antibiotics (Basel) 13.
22. Diggle SP, Whiteley M. 2020. Microbe Profile: Pseudomonas aeruginosa: opportunistic pathogen and lab rat. Microbiology (Reading) 166:30-33.
23. He Y, Li K-X, Wang J-W, Wang W, Fan P-C, Chen H-H, Wang J-J. 2021. Microbial Community Structure of Waste Water Treatment Plants in Different Seasons. Huan jing ke xue= Huanjing kexue / [bian ji, Zhongguo ke xue yuan huan jing ke xue wei yuan hui "Huan jing ke xue" bian ji wei yuan hui] 42:1488-1495.
24. Serletti L, Dutcher L, Degnan KO, Szymczak JE, Cluzet V, David MZ, Cressman L, Glassman LW, Hamilton KW, Centers for Disease C, Prevention Prevention Epicenters P. 2023. Analysis of seasonal variation of antibiotic prescribing for respiratory tract diagnoses in primary care practices. Antimicrob Steward Healthc Epidemiol 3:e147.
25. Velazquez-Meza ME, Galarde-López M, Cornejo-Juárez P, Bobadilla-Del-Valle M, Godoy-Lozano E, Aguilar-Vera E, Carrillo-Quiroz BA, Ponce de León-Garduño A, Velazquez Acosta C, Alpuche-Aranda CM. 2025. Bacterial Communities and Resistance and Virulence Genes in Hospital and Community Wastewater: Metagenomic Analysis. Int J Mol Sci 26.
26. Cai L, Ju F, Zhang T. 2014. Tracking human sewage microbiome in a municipal wastewater treatment plant. Appl Microbiol Biotechnol 98:3317-26.
27. Hendriksen RS, Munk P, Njage P, van Bunnik B, McNally L, Lukjancenko O, Röder T, Nieuwenhuijse D, Pedersen SK, Kjeldgaard J, Kaas RS, Clausen PTLC, Vogt JK, Leekitcharoenphon P, van de Schans MGM, Zuidema T, de Roda Husman AM, Rasmussen S, Petersen B, Bego A, Rees C, Cassar S, Coventry K, Collignon P, Allerberger F, Rahube TO, Oliveira G, Ivanov I, Vuthy Y, Sopheak T, Yost CK, Ke C, Zheng H, Baisheng L, Jiao X, Donado-Godoy P, Coulibaly KJ, Jergović M, Hrenovic J, Karpíšková R, Villacis JE, Legesse M, Eguale T, Heikinheimo A, Malania L, Nitsche A, Brinkmann A, Saba CKS, Kocsis B, Solymosi N, et al. 2019. Global monitoring of antimicrobial resistance based on metagenomics analyses of urban sewage. Nature Communications 10:1124.
28. Dekaboruah E, Suryavanshi MV, Chettri D, Verma AK. 2020. Human microbiome: an academic update on human body site specific surveillance and its possible role. Arch Microbiol 202:2147-2167.

29. Jiao Y-N, Zhou Z-C, Chen T, Wei Y-Y, Zheng J, Gao R-X, Chen H. 2018. Biomarkers of antibiotic resistance genes during seasonal changes in wastewater treatment systems. Environmental pollution (Barking, Essex : 1987) 234:79-87.
30. Lee CR, Lee JH, Park M, Park KS, Bae IK, Kim YB, Cha CJ, Jeong BC, Lee SH. 2017. Biology of Acinetobacter baumannii: Pathogenesis, Antibiotic Resistance Mechanisms, and Prospective Treatment Options. Front Cell Infect Microbiol 7:55.
31. Gómez P, Lozano C, Benito D, Estepa V, Tenorio C, Zarazaga M, Torres C. 2016. Characterization of staphylococci in urban wastewater treatment plants in Spain, with detection of methicillin resistant Staphylococcus aureus ST398. Environ Pollut 212:71-76.
32. Ben Said M, Abbassi MS, Gómez P, Ruiz-Ripa L, Sghaier S, Ibrahim C, Torres C, Hassen A. 2017. Staphylococcus aureus isolated from wastewater treatment plants in Tunisia: occurrence of human and animal associated lineages. J Water Health 15:638-643.
33. Bengtsson-Palme J, Hammarén R, Pal C, Östman M, Björlenius B, Flach CF, Fick J, Kristiansson E, Tysklind M, Larsson DGJ. 2016. Elucidating selection processes for antibiotic resistance in sewage treatment plants using metagenomics. Sci Total Environ 572:697-712.
34. Yang Y, Li B, Ju F, Zhang T. 2013. Exploring variation of antibiotic resistance genes in activated sludge over a four-year period through a metagenomic approach. Environ Sci Technol 47:10197-205.
35. Karkman A, Johnson TA, Lyra C, Stedtfeld RD, Tamminen M, Tiedje JM, Virta M. 2016. High-throughput quantification of antibiotic resistance genes from an urban wastewater treatment plant. FEMS Microbiol Ecol 92.
36. Wen Q, Yang L, Duan R, Chen Z. 2016. Monitoring and evaluation of antibiotic resistance genes in four municipal wastewater treatment plants in Harbin, Northeast China. Environ Pollut 212:34-40.
37. Pärnänen KMM, Narciso-da-Rocha C, Kneis D, Berendonk TU, Cacace D, Do TT, Elpers C, Fatta-Kassinos D, Henriques I, Jaeger T, Karkman A, Martinez JL, Michael SG, Michael-Kordatou I, O'Sullivan K, Rodriguez-Mozaz S, Schwartz T, Sheng H, Sørum H, Stedtfeld RD, Tiedje JM, Giustina SVD, Walsh F, Vaz-Moreira I, Virta M, Manaia CM. 2019. Antibiotic resistance in European wastewater treatment plants mirrors the pattern of clinical antibiotic resistance prevalence. Sci Adv 5:eaau9124.
38. Rumky J, Kruglova A, Repo E. 2022. Fate of antibiotic resistance genes (ARGs) in wastewater treatment plant: Preliminary study on identification before and after ultrasonication. Environ Res 215:114281.
39. Liu Z, Klümper U, Liu Y, Yang Y, Wei Q, Lin JG, Gu JD, Li M. 2019. Metagenomic and metatranscriptomic analyses reveal activity and hosts of antibiotic resistance genes in activated sludge. Environ Int 129:208-220.

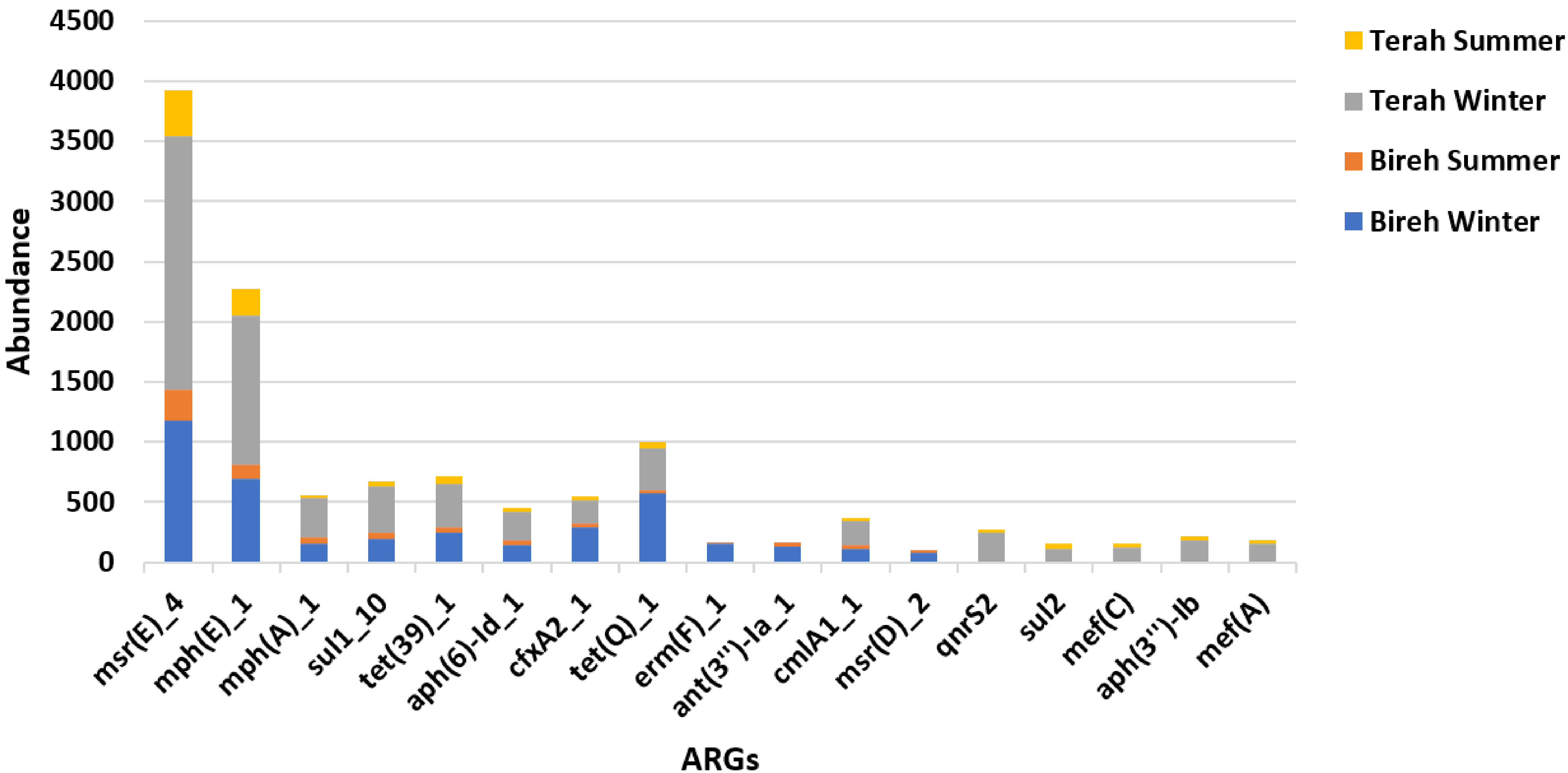

Figure 1: Difference in abundance (No. of reads) of ARGs between winter and summer found in influent samples taken from Al-Bireh and Al-Terah WWTPs

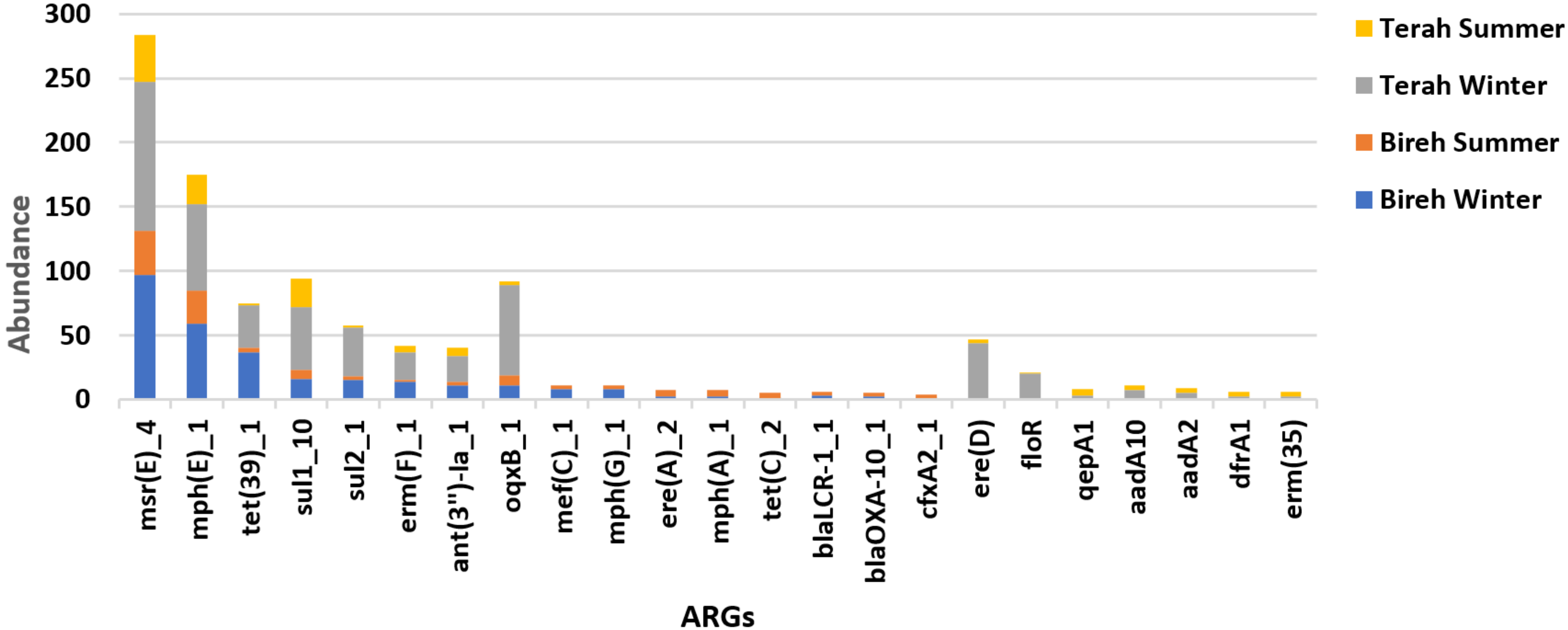

Figure 2: Differences in abundance (No. reads) of ARGs in winter compared to summer in effluent samples from Al-Bireh and Al-Terah WWTPs

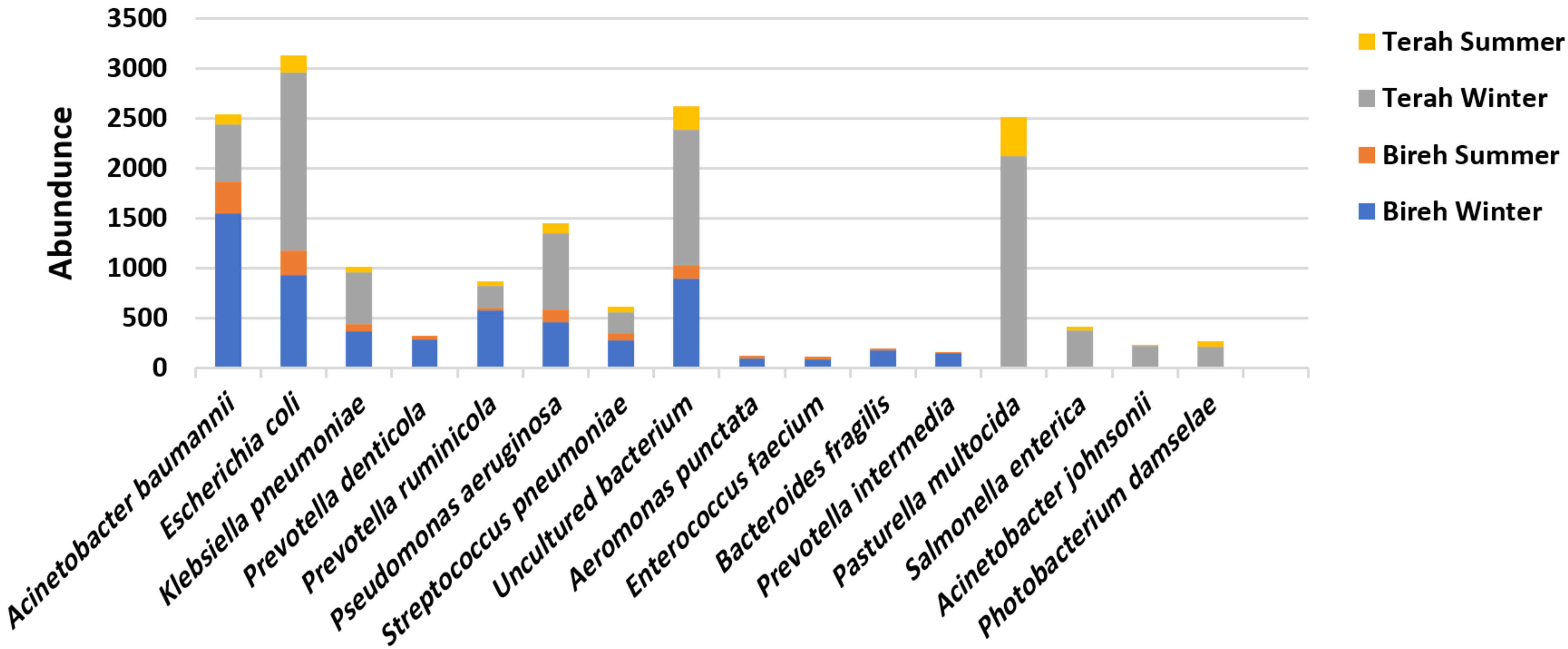

Figure 3: Differences in abundance (No. of reads) of major ARB species between winter and summer in influent samples taken from Al-Bireh and Al-Terah WWTPs

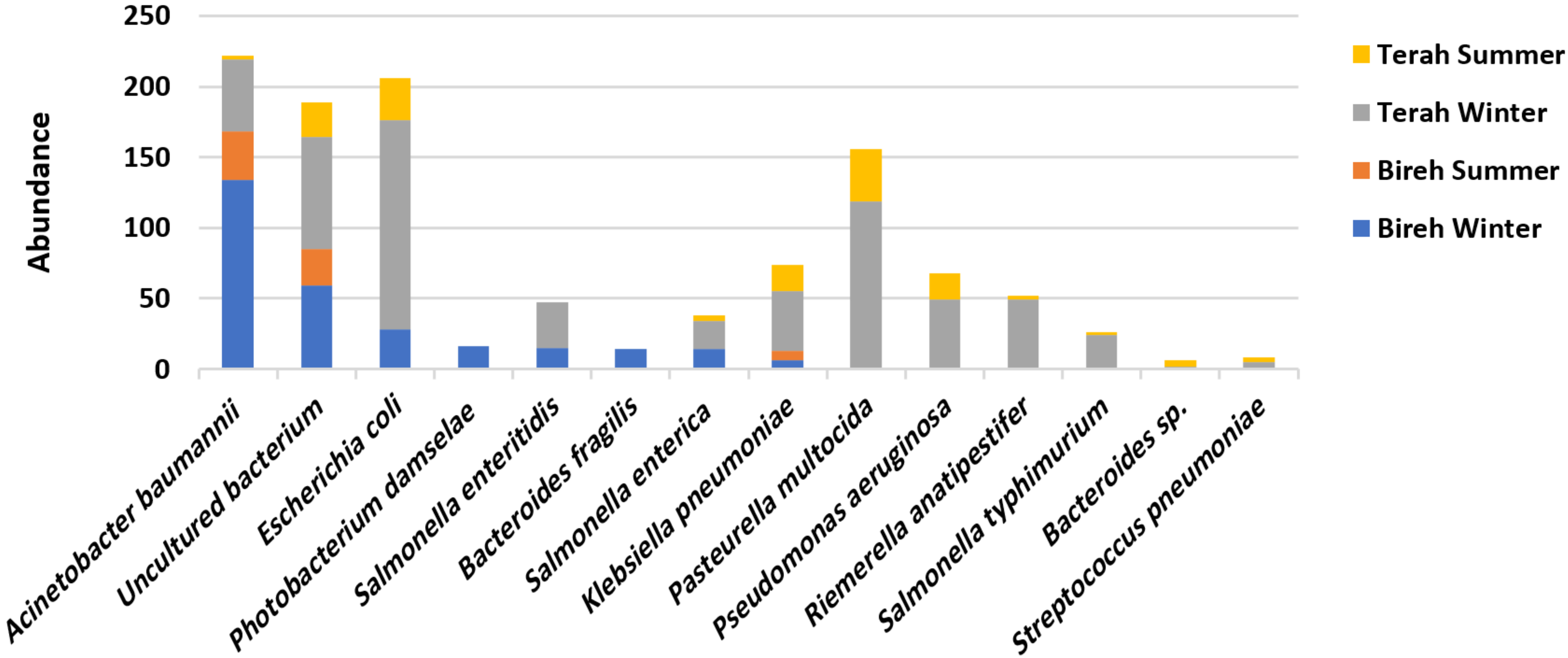

Figure 4: Difference in abundance of the major bacterial species (No. of reads) between winter and summer in effluent samples from Al-Bireh and Al-Terah WWTPs.

Comments for the Author

Reviewer #1

The manuscript by Hroub M. et al. used metagenomic sequencing to profile antibiotic-resistant bacteria (ARB) and antibiotic resistance genes (ARGs) and to examine their seasonal variation in two Palestinian wastewater treatment plants (Al-Bireh and Al-Terah). By comparing influent and effluent samples collected in winter and summer, the study provided an overview of microbial community composition and the apparent removal of these contaminants during treatment. The authors showed greater ARG diversity and higher ARB abundance in winter, and identified Acinetobacter baumannii and Pasteurella multocida as the predominant taxa.

Although this research topic is of potential to the field of public health and environmental microbiology, I have some concerns regarding the methodology and the interpretations of some key findings.

Major concerns:

1.Bofinformatic workflow (lines 154-166) is not very clear to me and not sufficiently described, which influences my comments on this manuscript. In particular, the authors should clearly describe the read-level quality control steps (e.g., adapter removal, quality filtering, removal of low-complexity reads and potential contaminants), and report relevant QC metrics. In addition, it is unclear whether metagenomic assembly was performed. If assembly was used, the authors should specify the assembler, version, key parameters, minimum contig length thresholds, and provide assembly statistics (e.g., total assembly size, N50, number of contigs). Without these details, the ARG detection and quantification strategy cannot be properly assessed.

We have used online Galaxy plat for the bioinformatic analysis. A study limitations paragraph has been added at the end of 'Discussion' section prior to conclusion.

2.I am not well convinced about the approach that the authors used for assigning bacterial host to ARGs. The manuscript appears to predict bacterial hosts by BLASTing GenBank accession numbers associated with ARG references. This approach is not appropriate for host attribution in metagenomic samples, because ARGs are frequently carried on mobile genetic elements (e.g., plasmids, integrons, transposons) and can occur in multiple taxa. Therefore, database associations do not demonstrate which organisms carry these genes in the sampled wastewater. The current approach is likely

to over-interpret database co-occurrence as true host assignment. More defensible approaches include linking ARGs to assembled contigs with taxonomic classification, MAG-based binning followed by ARG annotation, or clearly presenting the results as "known/potential carriers" rather than confirmed hosts.

A table with Accession number and frequencies were added to Appendix 2(f). It is not possible to make further data modification as part of the work was done elsewhere that became unreachable after the turmoil in the Middle East. More explanation was added on the assembly (Quality control and assembly)

3.I have a big concern about how removal efficiency is calculated and what quantitative measure is used (e.g., frequency, hit counts, raw read counts). Because sequencing depth varies substantially among samples (especially between influent and effluent), the authors must normalize the data (e.g., reads per million, RPKM/TPM, or coverage-based measures) before computing removal percentages. Otherwise, the estimated "removal efficiency" may primarily reflect differences in sequencing depth rather than true treatment performance.

A sentence was added to indicate that removal efficiency was based on the implementing the equation for the number of reads for ARB and ARG.

Minor comments:

1.Please include a map showing the WWTP locations, sampling points and relevant information if necessary.

Two maps were added as Appendix figure 1.

2.Line 151: Please specify the read-length.

Changed to show the sequence size as 150 bp

3.Line 178: Please carefully use "deep metagenomic" words. Some samples have very low read counts; consider avoiding "deep" unless supported by coverage analyses.

The word ‘deep’ was deleted.

4.Line 164: "the statistical package SPSS v29 (IBM Corporation, USA)....". It seems that the description of statistical analyses is incomplete. Please specify which comparisons were tested (season, influent vs effluent, WWTP differences), the statistical tests used, and any corrections for multiple comparisons, if applicable.

This is all explained in details in appendices 1 and 2 (Excel files)

5.Please provide accession numbers for raw reads.

A table with Accession number and frequencies were added (Appendix 2(f)).

Reviewer #2

This study investigates antibiotic resistance genes (ARGs) in the West Bank, Palestine, by looking at four different wastewater treatment plants via metagenomics profiling. The authors provide conclusions on the prevalence of ARGs and the microbial species they come from across these plants and also by samples collected in February and August. The study presents a great dataset, however the bioinformatic processing is unclear and the results need to be presented in a normalised way or explained clearly. I have proposed some additional analyses below. Given this, it is hard for me to fully assess the conclusions/discussion at this time without further clarification and/or analyses.

Major Concerns

The bioformatics pipeline requires some additional information. Can you full describe the galaxy pipeline that was used, what programmes/algorithms are run for assembly, trimming, quality check, etc? In addition please report metrics to inform the quality of the sequencing and assembly. Such as number of contigs per sample, the N50, L50, etc. Can you also state the number of reads that were in the influent and effluent after read trimming, or are the values reported in Table 1 after trimming? **A new paragraph 'Quality control and assembly' has been added in which trimming was processed using Trimmomatic tool in Galaxy software.**

In additional I recommend to run reads mapping of the samples back to ARGs you have identified to provide some quantitate information, as the number of ARGs indicate diversity but not necessarily how present they are. If you used reads mapped data and compared normalized (you could use a housekeeping gene) you would have a better idea of prevalence than just number of ARGs. I suggest to keep the information provided in Figures 1 and 2, but maybe add a second panel to the figure w/ the normalised reads mapping data. **It is not possible to make further data modification as part of the work was done elsewhere that became unreachable after the turmoil in the Middle East. Instead, we have added a table with Accession number and frequencies (Appendix 2(f)).**

I suggest to provide information on the predicted organisms that these ARGs are hosted by via contigs with ARGs identified. Was this done? Or were the ARG genes identified

and checked taxonomically without consideration to the rest of the contigs/genome/partial genomes assembled. If you only assign taxonomy based on the ARG itself you will miss out on information regarding horizontal gene transfer, and the actual origin of each ARG. In relation to human health some microbes are more relevant than others, identifying those that have ARGs would provide more impact to the study. This would go well with the taxonomic results. Additionally, what programmes/databases were used to assess taxonomy, I do not see this included in the methods. **A paragraph was added to clarify the processing of reads. Appendix 2 table has been modified to include ARG frequencies with their Accession numbers as well as having the ARGs with the corresponding bacteria in both seasons.**
Are the reads for Figure 3 and 4 normalised somehow? I do not see this information in the methods so it is unclear how much influence sequencing depth is having on these comparisons. Plese explain this in the methods. **A control paragraph was added.**
The discussion could be strengthened by the support of the methods detailed above. **A limitation paragraph was added. More elaboration on the NGS work was mentioned in detail in our previous NGS work such as (Nasereddin et al., 2022)**

Minor Concerns

Are the "unculturable bacteria" truly unculturable or just not cultured at present? Are these just species discovered via sequencing and not yet grown in lab? I think the distinction is important. This is throughout the manuscript. **Unculturable bacteria is a common term used when working with environmental samples as they do not grow on standard microbiology media like 5%SBA, MacConkey, chocolate, etc. They may grow under special conditions or can be detected by the presence of their DNA print. A sentence was added in the introduction to clarify the idea.**

Line 57 - is to are. **Corrected**
Line 60 humans "and" animals. **Corrected**
Line 77 sentence needs restructuring **Restructured**
Line 79 the to a **Corrected**
Line 88, please characterise the seasons further with metadata for the sampling days. Coordinates, temperature, weather, etc - anything you have. Coordinate and dates are critical. **Data on temperature, annual rain fall and coordinates were added in a new paragraph.**
Line 100 47500, remove the period **Corrected**
Line 123, should "collected" be combined into one tube ? **Corrected**
Please include the tapestation results in supplemental, mentioned at line 149.

Unavailable at this time in the Middle East
Line 155 fastaq to fastq **Corrected**

Please upload your raw data to a public platform and provide an accession number. **All Accession numbers were added to Appendix 2 (f)**

Re: Spectrum00028-26R1 (Metagenomic Identification of Antibiotic Resistance Genes and Bacterial Hosts in Wastewater Treatment Plants in Palestine)

Dear Prof. Amer Al-Jawabreh:

Thank you for addressing all of the comment's of the previous reviewers, who have had an opportunity along with myself to review the revised manuscript. I am pleased to inform you that your manuscript has been accepted, and I am forwarding it to the ASM production staff for publication. Your paper will first be checked to make sure all elements meet the technical requirements. ASM staff will contact you if anything needs to be revised before copyediting and production can begin. Otherwise, you will be notified when your proofs are ready to be viewed.

Data Availability: ASM policy requires that data be available to the public upon online posting of the article, so please verify all links to sequence records, if present, and make sure that each number retrieves the full record of the data. If a new accession number is not linked or a link is broken, provide production staff with the correct URL for the record. If the accession numbers for new data are not publicly accessible before the expected online posting of the article, publication may be delayed; please contact ASM production staff immediately with the expected release date.

Publication Fees: For information on publication fees and which article types have charges, please visit ourwebsite. We have partnered with Copyright Clearance Center (CCC) to collect author charges. If fees apply to your paper, you will receive a message from no-reply@copyright.com with further instructions. For questions related to paying charges through RightsLink, please contact CCC at ASM_Support@copyright.com or toll free at +1-877-622-5543. CCC makes every attempt to respond to all emails within 24 hours.

ASM Membership: Corresponding authors may join or renew ASM membership to obtain discounts on publication fees. Need to upgrade your membership level? Please contact Customer Service at Service@asmusa.org.

Embargo Policy: A press release may be issued as soon as the manuscript is posted on theSpectrum Latest Articles webpage. The corresponding author will receive an email with the subject line "ASM Journals Author Services Notification" when the article is available online.

Cover Image Submissions: If you would like to submit a potential Cover Image, please email a file and a short legend to spectrum@asmusa.org. Please note that we can only consider images that (i) the authors created or own and (ii) have not been previously published. By submitting, you agree that the image can be used under the same terms as the published article. Image File requirements: TIF/EPS, 7.5 inches wide by 8.25 inches tall (at least 2,250 pixels wide by 2,475 pixels tall), minimum 300 dpi resolution (600 dpi preferred), RGB, and no figure elements, e.g., arrows or panel labels. The legend should be a short description of the image, 1-2 sentences recommended. Please download and use this interactive template in Adobe the https://journals.asm.org/pb-assets/html/asm-cover-sizing-tool.html to ensure that your proposed cover image meets our size requirements (https://journals.asm.org/pb-assets/pdf-text-excel-files/ASM-Interactive-Sizing-Cover-Template-1715689791.pdf).

The ASM Journals program strives for constant improvement in our submission and publication process. Please tell us how we can improve your experience by taking this quick Author Survey.

Thank you for submitting your paper to Spectrum.

Sincerely,
Michael Becker
Editor
Microbiology Spectrum

Reviewer #1 (Comments for the Author):

Dear authors, thank you for answering all the remarks requested by the reviewers in a mostly scientific way. Thank you

Metagenomic Identification of Antibiotic Resistance Genes and Bacterial Hosts in Wastewater Treatment Plants in Palestine

Mosab Hroub[1], Suheir Ereqat[2], Amer Al-Jawabreh[3,4*], and Ziad Abdeen[5]

1 Hebron University, Al-Khalil, Palestine

2 Biochemistry and Molecular Biology Department, faculty of Medicine, Al-Quds University, Jerusalem, Palestine

3 Leishmaniases Research Unit-Jericho, Palestine

4. Arab American University, Palestine

5. Al-Quds Public Health Society, Palestine

***** Corresponding author: Al-Jawabreh A.; islahjr@yahoo.com, amer.aljawabreh@aaup.edu; Jericho, Palestine

ABSTRACT

Wastewater treatment plants (WWTPs) are recognized as significant sources for the dissemination of antibiotic-resistant bacteria (ARB) and antibiotic resistance genes (ARGs). In the West Bank, Palestine, four WWTPs are currently working in Jenin, Tulkarem, Ramallah (Al-Terah), and Al-Bireh, most of which use secondary treatment processes based on activated sludge. In this study, Illumina high-throughput sequencing was used to characterize the profiles of ARB and ARGs in the Al-Bireh and Al-Terah WWTPs. Raw wastewater (influent) and secondary-treated water (effluent) samples were collected during two seasons, summer and winter. The results revealed a higher abundance of ARB and greater diversity of ARGs in winter compared to summer. The most dominant species detected were *Acinetobacter baumannii* in Al-Bireh samples and *Pasteurella multocida* in Al-Terah samples. In total, 138 distinct ARGs providing resistance to 12 antibiotic classes were identified, with genes providing resistance to macrolides and tetracyclines were the most prevalent. The removal efficiency of the ten most common ARB and ARGs ranged from 85% to 100% in both treatment plants. In conclusion, unfiltered antibiotics and ARGs in treated wastewater can be absorbed by plants and crops, posing a potential public health risk. The abundance

of ARGs and their bacterial hosts was higher in winter, highlighting the need to enhance sanitation measures and improve treatment efficiency. Strengthening wastewater treatment systems should be a priority for policymakers to prevent the spread of ARB and ARGs in Palestine.

IMPORTANCE Untreated wastewater plays a key role in spreading antibiotic-resistant bacteria (ARB) containing antibiotic-resistant genes (ARG) in the environment. The current study characterized the ARB and ARG in two wastewater treatment plants (WWTP) in Jenin and Ramallah, Palestine in summer and winter in both pre-treated and post-treated water. In winter, ARB were more abundant, and ARG were highly diverse in both plants alike. *Acinetobacter baumanii*, an extremely drug-resistant bacterium, was found as a dominant bacteria. The study revealed 12 ARG groups based on the antibiotic class in the two WWTP, with β-lactamases showing the highest number of resistant genes. The efficiency of removal of bacteria in both plants ranged from 85-100%.

KEYWORDS: ARGs, ARB, MDR, wastewater treatment, activated sludge, influent, effluent

INTRODUCTION

Wastewater treatment (WWT) is a process designed to remove pollutants and contaminants from wastewater (WW) through biological, chemical, and physical methods. Once treated, the wastewater can be safely discharged into the environment. Sludge, a by-product of the treatment process derived from sewage, requires further processing before reuse or disposal. It consists of two types: sludge and activated sludge, both containing various organic and inorganic compounds. Sludge has a high water content originating from liquid wastewater with a small proportion of solid waste. It can be a primary sludge that produced during the primary treatment stage, or secondary sludge, resulting from the secondary treatment process. Activated sludge (AS) contains different types of microorganisms that live in harmony together. The microbial diversity in AS is higher than that in incoming wastewater (IW) and includes many uncultivable species. The main difference between sewage and

sludge is that sewage is a suspension of solid and liquid waste, whereas sludge refers to the solid material separated from that suspension (1).

The WW treatment process consists of three main stages: the physicochemical process as the primary step, the biological process as the secondary step, and a tertiary process that involves advanced methods such as the advanced oxidation process. The main objective of these steps is to produce high-quality water suitable for reuse in various applications (2).

A huge diversity of pathogenic and nonpathogenic microorganisms is found living together in wastewater, sharing metabolic activities to support the growth and multiplication of one another. The WWTPs became a hotspot for the presence of microorganisms, especially the antibiotic-resistant organisms that come from humans /animals and are released into sewage through feces, urine, dead bodies, and manure. Moreover, the WW that flows from hospitals and farming facilities could be the major source of antibiotic resistance genes (ARGs) and antibiotic-resistant bacteria (ARB) that are released in the environment (3).

The main route of transfer of resistance in the AS occurs by horizontal transfer of ARGs through mobile genetic elements (MGEs) such as plasmids, transposons, bacteriophages, insertion sequences, and integrons(4). There are many ARGs that encode for resistance against different antibiotics like tetracyclines (tet), sulfonamides (sul), β-lactams (bla), macrolides (erm), aminoglycosides (aac), fluoroquinolone (fca), colistin (mcr), vancomycin (van), and multidrug (mdr) (5). It was shown that MGEs were abundant in the sludge samples, and this may be important for the acquisition and mobility of various ARGs among the bacterial species (6). The spreading of ARGs and ARB in the environment could lead to antibiotic resistance in humans and threaten public health (3).

In the past, culture-based identification methods faced challenges in analyzing microbial communities from environmental samples, as they relied mainly on morphological characteristics for microorganism identification. Additional limitations of these methods include the low selectivity of

culture media and the need for diverse culture conditions, which often inhibit or prevent the growth of approximately 99% of the bacterial community present in wastewater (7, 8).

On the other hand, molecular methods are benefiting in the determination of microorganisms and their biological activity under different situations. They allow the real-time observation of the microorganisms and their metabolic pathways. Accordingly, the shotgun metagenomic method is capable of determining the whole genomic DNA in environmental samples without the need to isolate or culture individual microorganisms in the laboratory(9). This contrasts with the classical PCR method, which depends on specific primers to isolate ARGs and detect their abundance in the bacteria. Moreover, classical PCR is limited in detecting novel ARGs compared to next-generation metagenomic analysis (10). Furthermore, the metagenomic approach can detect the low-abundance microorganisms by generating hundreds to thousands of sequences and give a clear picture of the microbial community found in the sample (11). Using metagenomic analysis, this study aimed to profile ARB and their associated antibiotic ARGs isolated from two WWTPs across two seasons, analyzing both pre- and post-treatment samples, and to evaluate the removal efficiency of the selected WWTPs in Palestine.

MATERIALS AND METHODS

WWTPs description

The water samples were collected from both Al-Bireh and Al-Terah WWTPs, which are located in Ramallah city. Al-Bireh plant was constructed in 1997 and started functioning in February 2000. It has a capacity of 6000 m^3/day of treated wastewater, and serves about 50000 people in Al-Bireh town. This WWTP depends on the conventional activated sludge method as a secondary treatment process, including a low-loaded activated sludge stage, which, besides the removal of carbonaceous compounds, it performs oxidation of nitrogen compounds (nitrification). The treatment process is performed in a unit composed of a mixing and distribution chamber for aeration tank feeding without the involvement of tertiary treatment process. However, the effluent output of this plant is released

into the wadies. Al-Terah plant located in Al-Terah town and serves 47.500 population. The capacity of this plant is 1370 m^3/day. It depend on two aeration tanks to treat wastewater. However, the treated water is deposited in Wadi Bitunia (12).

Sampling

Two different types of samples from both WWTPs were collected using sterile 500ml bottles (Duran, Inc. USA). From each WWTP, one raw (influent) and one treated (effluent) sample were collected in two different seasons: winter (February) and summer (August). The composite sampling involved taking equal subsamples every five meters in sterile bottles to finally have the whole sample to ensure representativeness, then transported within 48 hours in a cool box to the laboratory for DNA extraction.

Samples preparation

Influent samples (500ml) were first divided equally into ten samples using 50ml sterile Falcon tubes, then centrifuged at maximum speed, about 3220 g, for 20 minutes in a 5810 R centrifuge (Eppendorf, Germany) to obtain the pellet. The pellets were reconstituted with absolute ethanol in a ratio of 1:2 and kept in -20°C to be used for DNA extraction (Ma, Li et al. 2017).

The 500ml effluent samples were filtered using a Polyvinylidene difluoride (PVDF) membrane filter (Stericup 250 ml, Durapore, 0.45 μm PVDF). The membrane then then removed carefully using sterile scissors, and forceps, transferred to sterile tubes, and kept at -20°C to be used for DNA extraction.

DNA extraction and concentration

Influent samples

The 50ml frozen samples were thawed, homogenized separately by vortex mixer, and finally the ten tubes were collected in one tube. For DNA extraction, QIAamp® DNA Mini Kit (Qiagen GmbH, Germany) was used according to manufacturer’s instructions of bacterial DNA extraction protocols,

except in the final step, in which 50µl of elution buffer was added instead of 200µl to obtain a final extract volume of 50µl.

Effluent samples

The filter papers were thawed, carefully removed from the tube, and cut into small pieces using sterile scissors. The DNA was extracted from filter papers using the same kit and the same procedures used for influent samples. The concentration of DNA samples was measured using the Qubit v4 (Invitrogen, USA) machine. DNA concentration was adjusted to 0.4 ng/µl to be accepted for library preparation.

Library preparation

For metagenomic analysis, the DNA library was prepared using the Nextera XT DNA library preparation kit (illumina®, USA). Briefly, the gDNA was normalized to have five microliters of DNA. Tagmentation was done using five µl of Tagmentation buffer (Amplicon Tagment Mix) with a special Tagmentation mixture that contains transposomes and then incubated at 55℃ for five minutes. Five µl of Neutralize Tagment Buffer (NT) was added to the mix and incubated for five minutes at room temperature. Two different index adapters were added (five microliters each) at both ends of the DNA fragment. To attach those index adapters to the DNA, a 15µl of Nextra PCR master mix were added to 35µl of tagmented DNA to a final volume of 50µl, the reaction mixture was subjected to thermal cycling as follows: 72℃ for 3 minutes, 95℃ for 30 seconds, then 12 cycles of 95℃ for 10 seconds, 55℃ for 30 seconds, 72℃ for 30 seconds, then 72℃ for 5 minutes, and finally put on hold at 10℃. The obtained DNA library with fragments between 300-500bp was then purified with 1X AMPure XP beads (Beckman Coulter, Brea, CA, USA). The concentration of the prepared DNA library was measured using the Qubit v4 (Invitrogen, USA) machine. The quality of the library was evaluated by the Tapestation machine (Agilent, CA, USA), and the library was normalized to

four nmol. Sequencing (as a single read) was done using the NextSeq 500/550 High Output Kit v2.5 (150 Cycles).

Bioinformatics analysis

Analysis workflow

The obtained fastaq files were converted into Fasta format using the FASTQ to FASTA converter from the FASTX- toolkit command on the online Galaxy software (www.usegalaxy.org).. The annotation process was done using the ABRicate command, which performs mass screening of contigs for antimicrobial and virulence genes. The output results of this command included the related reference sequence which was indicated by the GenBank accession number. The accession number accompanied with each ARG from the previous Galaxy workflow analysis was BLAST searched (www.blast.ncbi.nlm.nih.gov) to identify the bacterial host. Each organism with an identity and query cover above 97% was included (13). After downloading the results file of the ABRicate, the statistical package SPSS v29 (IBM Corporation, USA) was used to measure the frequency of each ARGs. Then ‘Cross Tab’ command was applied to link the accession number to different types of ARGs (Appendix 2).

ARGs analysis

AMR gene family, drug class, and resistance mechanisms for each gene were analyzed using ‘The Comprehensive Antibiotic Resistance Database (CARD, https://card.mcmaster.ca/), The ‘Analyze’ command on the CARD main page was chosen, then the reference gene identifier (RGI) was selected to search for resistant genes using the GenBank accession numbers obtained from the previous Galaxy workflow analysis.

Removal efficiency

The removal efficiency of ARGs was calculated by the following equation described elsewhere (14).

$$\%\text{Removal Efficiency} = \frac{\text{Influent frquency}}{\text{Influent freq} - \text{Effluent freq}} \text{x}100\%$$

RESULTS

Our study utilized a deep metagenomic sequencing approach to analyze the ARGs present in influent and effluent samples collected from both the Al-Bireh and Al-Terah wastewater treatment plants across two different seasons. During winter, the total number of sequencing reads was higher in Al-Terah WWTP than in Al-Bireh for both influent and effluent samples. Conversely, in summer, Al-Bireh exhibited a greater number of reads than Al-Terah in both sample types. (Table 1).

Table 1. The overall metagenomics characteristics in the two WWTPs, Al-Bireh and Al-Terah, in winter and summer

	Al-Bireh WWTP		Al-Terah WWTP	
	Winter	Summer	Winter	Summer
No. reads, influent	$3.7x10^{6}$	$0.37x10^{6}$	$5.09x10^{6}$	$0.031x10^{6}$
No. reads, effluent	$0.095x10^{6}$	$0.26x10^{6}$	$0.32x10^{6}$	$0.022x10^{6}$
No. ARGs, influent	400	253	305	162
Prevalent ten, %	44	38	51	56
ARGs	(msr(E), mph(E), tet(Q), cfxA2, tet(39), sul1, erm(F), mph(A), tet(Q), aph(6)-Id)	(msr(E), mph(E), mph(A), sul1, aph(6)-Id, tet(39), cfxA2, ant(3")-Ia, cmlA1, nd msr(D)	(msr(E), mph(E), sul1, tet(39), tet(Q), mph(A), qnrS2, aph(6)-Id, cmlA1, and cfxA2	(msr(E), mph(E), tet(39), tet(Q), sul1, sul2, mef(C), cfxA2, aph(3")-Ib, and mef(A)
Antibiotics, influent	Macrolide, tetracycline, Beta-lactamase, sulfonamide, streptogramin , aminoglycoside	Macrolide, tetracycline, aminoglycoside, Sulfonamide Trimethoprim	Macrolide, Sulfonamide ,tetracycline, aminoglycoside, Chloramphenicol/florfenicol, Beta-lactamase Quinolone	Macrolide, Sulfonamide ,tetracycline, aminoglycoside, Beta-lactamase
No. ARGs, effluent	88	72	97	52

Prevalent ten, %	67	51	62	66
ARG subtypes	msr(E), mph(E), tet(39), sul1, sul2, erm(F), ant(3")-Ia, oqxB_, mef(C), and mph(G)	msr(E), mph(E), mph(A), sul1, ere(A), tet(C), cfxA2, ant(3")-Ia, blaOXA-10, blaLCR-1	msr(E), oqxB, mph(E), sul1, ere(D), sul2, tet(39), erm(F), ant(3")-Ia, and floR	msr(E), mph(E), sul1, ant(3")-Ia,erm(F),qepA1, aadA10, aadA2,dfrA1, erm(35)
Antibiotics, effluent	Macrolide, Tetracycline, Sulfonamide, streptogramin , aminoglycoside	Macrolide, Beta-lactamase, Sulfonamide tetracycline, aminoglycoside	Macrolide,Tetracyline, aminoglycoside, Chloramphenicol/florfenicol, sulfomamide	Macrolide,sulfonamide aminoglycoside,

ARGs abundance

In winter, 400 antibiotic resistance gene (ARG) subtypes were identified in the influent samples from the Al-Bireh WWTP. The ten most prevalent ARGs accounted for 44% of the total and, in order of high abundance, included msr(E), mph(E), tet(Q), cfxA2, tet(39), sul1, erm(F), mph(A), tet(Q), and aph(6)-Id (Fig. 1, Table 1). These ARGs conferred resistance to several antimicrobial drug families, predominantly macrolides and tetracyclines, followed by β-lactams, sulfonamides, streptogramins, and aminoglycosides. In the influent samples from the Al-Terah WWTP, 305 ARG subtypes were detected, with the ten most prevalent representing 51% of the total. These included msr(E), mph(E), sul1, tet(39), tet(Q), mph(A), qnrS2, aph(6)-Id, cmlA1, and cfxA2 (Fig. 1, Table 1). The ARGs in Al-Terah conferred resistance to antibiotic families similar to those in Al-Bireh, with some differences such as the presence of quinolone resistance genes, which were detected only in Al-Terah.

During summer, 253 ARG subtypes were identified in influent samples from Al-Bireh WWTP, with the ten most dominant ARGs comprising 38% of the total. These included msr(E), mph(E), mph(A), sul1, aph(6)-Id, tet(39), cfxA2, ant(3")-Ia, cmlA1, and msr(D). Similar to winter, macrolide resistance genes were the most abundant, followed by those conferring resistance to tetracyclines,

aminoglycosides, sulfonamides, and trimethoprim. In Al-Terah WWTP during summer, 162 ARG subtypes were detected, with the ten most prevalent accounting for 56% of the total. These were msr(E), mph(E), tet(39), tet(Q), sul1, sul2, mef(C), cfxA2, aph(3")-Ib, and mef(A). The resistance gene families identified were consistent with those found in the Al-Bireh samples.

Figure 1: Difference in abundance (No. of reads) of ARGs between winter and summer found in influent samples taken from Al-Bireh and Al-Terah WWTPs

In winter, the effluent samples from the Al-Bireh WWTP contained 88 ARG subtypes, with msr(E), mph(E), tet(39), sul1, sul2, erm(F), ant(3")-Ia, oqxB, mef(C), and mph(G) comprising 67% of the total ARGs (Fig. 2, Table 1). The most abundant ARG families were consistent with those detected in the influent samples. In contrast, the Al-Terah WWTP effluent contained 97 ARG subtypes, with msr(E), oqxB, mph(E), sul1, ere(D), sul2, tet(39), erm(F), ant(3")-Ia, and floR representing 62% of the total. These ARGs conferred resistance to antibiotic families similar to those found in the influent samples.

In summer, the effluent samples collected from the Al-Bireh and Al-Terah WWTPs revealed 72 and 52 ARG subtypes, respectively. The ten most prevalent ARGs accounted for 51% of the total in Al-Bireh and 66% in Al-Terah (Fig. 2, Table 1). The dominant ARGs in both plants were associated with resistance to macrolide, sulfonamide, tetracycline, and aminoglycoside antibiotic classes (Appendix 2).

Figure 2: Differences in abundance (No. reads) of ARGs in winter compared to summer in effluent samples from Al-Bireh and Al-Terah WWTPs.

Identification of ARGs bacterial hosts and removal efficiency

In winter, 53 and 100 different bacterial species were found in influent samples from Al-Bireh and Al-Terah WWTPs, respectively. Ten bacterial species were found to comprise 74% and 77% of the total species abundance in Al-Bireh and Al-Terah plants, respectively. Regarding phyla frequency, the most abundant phyla were *Pseudomonadota* (67.8%), *Firmicutes* (8.4%), and *Bacteroidota* (6.3%). Uncultivable bacteria represented 11.3 and 11% in Al-Bireh and Al-Terah WWTPs, respectively. According to bacterial genera and species, *Acinetobacter baumannii* had the highest frequency in Al-Bireh WWTP compared to *Pasteurella multocida* in Al-Terah WWTP. While in summer, the influent

Figure 3: Differences in abundance (No. of reads) of major ARB species between winter and summer in influent samples taken from Al-Bireh and Al-Terah WWTPs.

samples from Al-Bierh WWTP contained 30 different bacterial species, with the most prevalent ten comprising 81% of the total bacterial species. However, in the Al-Terah influent sample, 68 species were found, with the most frequent ten comprising 76% of the total. At the phylum level, the most abundant phyla were *Pseudomonadota* (72%), *Firmicutes* (12-13%), uncultivable bacterium (10-11%), and *Bacteroidota* (2%) in the influent samples from Al-Bireh and Al-Terah samples.

On the other hand, in winter effluent sample, only eight bacterial species were found in Al-Bireh and 52 in Al-Terah WWTPs. The most common phyla were *Pseudomonadota* (63.55%) and *Bacteroidota* (6.3%), while uncultivable bacteria represented 1.27% in both Al-Bireh and Al-Terah plants, respectively. In the Al-Terah plant, *Firmicutes* (5%) and *Actinobacteria* (7%) were additionally found. However, in summer, the effluent sample from Al-Bireh WWTP contained only two bacterial species: *A. baumannii* and K. pneumoniae, belonging to the phylum *Pseudomonadota*. In contrast, the Al-Terah effluent sample was shown to contain 21 bacterial species. The dominant phyla were *Pseudomonadota*

(58%) followed by *Firmicutes* (13 %), *Bacteroidota* (3 %), and *Actinobacteria* (2%), whereas uncultivable bacterium comprised 22 % (Appendix 2).

Figure 4: Difference in abundance of the major bacterial species (No. of reads) between winter and summer in effluent samples from Al-Bireh and Al-Terah WWTPs.

In the samples from Al-Bireh WWTP, some of the bacteria such as *Prevotella* species and *Streptococcus pneumoniae*, were cleared from the effluent sample, and thus the removal efficiency was considered 100%. The removal efficiency for the other bacteria was still high ranging from 67-100%. While in Al-Terah WWTP samples, only *Acinetobacter johnsonii* has been cleared completely, with the removal efficiency ranging from 63 to100%. (Appendix 1). The removal efficiency of ARGs ranged from 85-100% in Al-Bireh WWTP and 87-100% in Al-Terah WWTP.

There are 12 ARG classification groups based on the antibiotic class. The most diverse one that comes with different resistance genes is beta-lactamase with 34 different genes, followed by tetracycline and aminoglycoside, which have 20 and 18 genes, respectively. The other group was ordered according to the number of genes. (Appendix-2).

DISCUSSION

As antibiotics were used for many years to eliminate bacteria, which wide range of these bacteria have acquired resistance to antibiotics by horizontal and vertical gene transfer (HGT) and mutations (15). This has introduced antibiotic ARB and ARG on a large scale into the environment, becoming an emerging global health problem (3, 16, 17). This gives the reason for estimating the level of ARGs in the activated sludge that ends up in the environment. The study aimed to document the presence of various types of ARB and ARGs in raw influent wastewater (WW) and treated effluent WW collected in two different seasons, winter and summer, from two WWTPs. The sources of raw wastewater that flow

to those plants are houses, slaughterhouses, and different hospitals in the area. In this study, a difference was observed in the amount of bacterial DNA, as represented by the number of bacterial reads, between influent and effluent samples across the two seasons. The decreased number of reads from thousands of reads prior to treatment to a couple of hundred post-treatment may reflect, to some extent, the efficiency of the treatment plant. However, the remaining amount of ARG in effluent can still spread in the environment with high risk potential (18). On the other hand, the reason for the high amount of bacterial DNA in winter rather than summer is due to rainwater that carries most of the microorganisms in the sewer pipeline and goes directly to plants. Moreover, the prevalence of bacterial infection is usually higher in winter than summer, especially among vulnerable people with weak immunity, such as children and the elderly, who may overuse broad-spectrum antibiotics and thus developing resistant bacteria (18, 19). Also, most bacteria, including ARB, that cause gastrointestinal, respiratory tract, and urinary tract infections are shed into the sewage system. In addition, the overuse of antibiotics results in excretion of unmetabolized antibiotics into the environment, which with the different pressures of pollutants, causes the spread of ARGs among bacterial communities in the environment (20). In the Al-Bireh plant, the treatment process depends basically on activated sludge which uses bacteria to degrade the contaminants and thus the microbial community. While in Al-Terah depend mainly on two aeration lagoons to treat water.

In winter, more bacterial species (54,100) were detected in the influent samples than in summer samples (3,068) taken from both Al-Bireh and Al-Terah WWTPs, respectively. Most of the detected in the influent and effluent samples were gram-negative bacteria, including Salmonella enterica and Pseudomonas aeruginosa (Fig 3 and 4), the WHO priority pathogens (21). The detected ARB are the most common causing disease and may survive at a wide range of temperatures and pH, such as Pseudomonas aeruginosa, with a growth range between 4.0 and 42°C and pH of 4.5 to 9.0 (22). He and

colleagues (23) showed that the amount and diversity of bacteria in winter were more than in summer, which is explained by increasing the biomass of the reactor to maintain the effectiveness of wastewater treatment under cold temperature, besides the increase of antibiotic use during winter, which may increase the number of resistant bacteria (24). At the phylum level, the most abundant ARB found in both seasons were *Pseudomonadota*, followed by *Firmicutes* and *Bacteroidota*. Of the *Pseudomonadota*, several significant species were identified, such as *Acinetobacter baumannii*, *Escherichia coli*, *Pasturella multocida*, and K. *pneumoniae*. However, these species were significantly more frequent in winter than in summer. Our results are consistent with several other studies (8, 25) and inconsistent with Ju et al study which reported that the most abundant phylum in the influent sample was *Firmicutes*, followed by *Pseudomonadota*, *Actinobacteria*, and *Bacteroidota* (26). In regards to genera frequencies, our results were in agreement with those reported by other studies (27), which showed that the most abundant bacterial genus across the samples was *Acinetobacter*, and disagreed with other results stating that the most frequent genera were Bacteroides, *Escherichia*, *Streptococcus* (27). Ahmed et al reported that the most dominant bacterial genera in raw wastewater samples were Pseudomonas, followed by Arcobacter and Bacteroides (8). In our study, all the bacterial species were found to match the pattern of human pathogens which cause different infections in the respiratory tract, gastrointestinal, and urinary tracts (28).

The effluent samples contained eight and 52 species in the winter sample compared to three and 21 species in the summer samples from Al-Bireh and Al-Terah, respectively, which is runs parallel to the distribution of ARGs. Most of them belonged to the bacterial phyla Pseudomonadota, followed by Bacteroidota, with lower relative abundance than the influent samples. Our results, inconsistent with another study (29) showed that the most abundant phylum Pseudomonadota was less frequent in winter than in summer. In the Al-bireh sample, *Acinetobacter baumanaii*, a commonly known multidrug-

resistant (MDR) *Proteobacteria* responsible for hospital-acquired infection (HAI), had the highest frequency in both seasons, while other studies showed that *Pseudomonas aeruginosa*, another MDR-HAI bacteria, was the most frequent. (8, 27, 30). In contrast, in Al-Terah samples, *Pasteurella multocida*, an animal-related opportunistic commensal *Pseudomonadota*, was found at high frequency. There were also some bacteria explored that pose a significant health impact, but not in the top 10 bacteria. Staphylococcus aureus (S. aureus) is considered to be an opportunistic pathogen that causes infection. Methicillin-resistant *Staphylococcus aureus* (MRSA) is the most significant type because it harbors different ARGs. S. aureus harbors different ARGs like msr(A), Inu(B), and tet(M), similar to our finding (31, 32).

In this study, we noticed that most of the bacteria were efficiently removed after treatment, which could be attributed to the high performance of plants, especially in the Al-Bireh than Al-Terah plant. Al-Bierh WWTPs it is based on using an aeration tank and activated sludge as a secondary process under the effect of oxygen, and the other uses an aeration lagoon, which depends on the oxygen pumped and circulated to build aerobic conditions to treat water. Moreover, it relies on a sedimentation process in which the pathogens are adsorbed in the biosolid phase (8). A dramatic decrease in the anaerobic bacteria was noted after treatment, which may be due to shifting from an anaerobic to aerobic conditions, replacing the anaerobic bacteria with facultative anaerobic ones with the aid of low temperature, and thus bacteria that live under oxygen condition will proliferate and be retained in the plant (33).

In the influent samples collected from Al-Bireh and Al-Terah, 400,305 ARGs subtypes were found in the winter samples compared to 253,162 ARGs subtypes found in summer. On the other hand, the effluent samples from the two plants, Al-Bireh and Al-Terah, contained effectively less ARGs than in the influent samples, with higher abundance of ARGs in summer (88 and 97, respectively) than in winter

(77 and 52, respectively) (Table 1, Appendix 2). The 1.6-fold ARG increase in winter could be explained by intensive use of antibiotics (34). However, a few studies showed that temperature differences between seasons have very little or no impact on ARG abundance (35, 36). Our study showed that the most abundant resistant genes in the two were msr(E) and mph(E), and to a lesser degree tet(Q). These genes were reported as macrolide resistance genes, especially to erythromycin antibiotics, including azithromycin, clarithromycin, and spiramycin (Fig. 3 and 4) which is in congruence with other studies (27, 34, 37). Several studies revealed different patterns of predominating resistant genes such as tet_Q, tetA, tetX (tetracycline), ermB (erythromycin), sul1 (sulfonamide), blaCTX-M (cefotaxime), and mecA (methicillin) (8, 38). The discrepancy in ARG profiles between studies can be explained by the different wastewater sources and treatment processes of WWTPs, the class of antibiotics consumed by the served community, and the completeness of ARG databases that rely on the depth of metagenomic sequencing (39).

Conclusion

Shotgun metagenomic analysis offers a clear advantage over classical PCR methods by enabling the detection of uncultivable bacteria and the identification of novel ARGs without targeting specific bacterial sequences. In this study, several pathogenic bacterial species were identified, including *Acinetobacter baumannii*. The abundance of ARGs and their associated bacterial hosts was higher in February compared to August. Although the overall removal efficiency in the studied wastewater treatment plants was high, the persistence and dissemination of unfiltered ARB and ARGs into the environment remain a major concern. These residual contaminants can infiltrate groundwater, surface water, soil, and crops, posing a significant threat to public health. Therefore, it is crucial to raise public awareness about the risks associated with wastewater containing high levels of pathogens and antibiotic resistance determinants. In addition, enhancing sanitation infrastructure and improving the efficiency of

wastewater treatment systems should be a priority for policymakers to mitigate the spread of ARB and ARGs in treated wastewater in Palestine.

ACKNOWLEDGMENT

This work was partially supported by the U.S Department of State under the identifier SIS70022GR0033 grant - Pharmaceuticals M35-018.

DATA AVAILABILITY

All data are available as appendix files.

REFERENCES

1. Poblete IBS, Araujo OdQF, de Medeiros JL. 2022. Sewage-Water Treatment and Sewage-Sludge Management with Power Production as Bioenergy with Carbon Capture System: A Review. Processes 10:788.
2. Fernandes J, Ramísio PJ, Puga H. 2024. A Comprehensive Review on Various Phases of Wastewater Technologies: Trends and Future Perspectives. Eng 5:2633-2661.
3. Garcia-Torné M, Falcó I, Borrell X, Bautista A, Mazigh R, Aznar R, Sánchez G, Farré M, Llorca M. 2025. Comprehensive Study of Antibiotics and Antibiotic Resistance Genes in Wastewater and Impacted Mediterranean Water Environments. Antibiotics 14:341.
4. Yu Z, Liu Z, Sun L, Dong C, Jin Y, Hu B, Cheng D. 2024. Mobile genetic elements mediate the cross-media transmission of antibiotic resistance genes from pig farms and their risks. Science of The Total Environment 926:172115.
5. Jian Z, Zeng L, Xu T, Sun S, Yan S, Yang L, Huang Y, Jia J, Dou T. 2021. Antibiotic resistance genes in bacteria: Occurrence, spread, and control. Journal of Basic Microbiology 61:1049-1070.
6. Guo J, Li J, Chen H, Bond PL, Yuan Z. 2017. Metagenomic analysis reveals wastewater treatment plants as hotspots of antibiotic resistance genes and mobile genetic elements. Water Research 123:468-478.
7. Hladilek MD, Gaines KF, Novak JM, Collard DA, Johnson DB, Canam T. 2016. Microbial community structure of a freshwater system receiving wastewater effluent. Environ Monit Assess 188:626.
8. Ahmed W, Staley C, Sidhu J, Sadowsky M, Toze S. 2016. Amplicon-based profiling of bacteria in raw and secondary treated wastewater from treatment plants across Australia. Applied microbiology and biotechnology 101:1253-1266.
9. Chen Y, Fan LC, Chai YH, Xu JF. 2022. Advantages and challenges of metagenomic sequencing for the diagnosis of pulmonary infectious diseases. Clin Respir J 16:646-656.
10. Schmieder R, Edwards R. 2012. Schmieder R, Edwards R.. Insights into antibiotic resistance through metagenomic approaches. Fut Microbiol 7: 73-89. Future microbiology 7:73-89.
11. Ahn Y, Choi J. 2016. Bacterial Communities and Antibiotic Resistance Communities in a Full-Scale Hospital Wastewater Treatment Plant by High-Throughput Pyrosequencing. Water 8:580.
12. Y. Mogheir TAH, Z. Zomlot , A. Ahmed and D. Fatta. 2005. Treated Wastewater Reuse in Palestine.
13. Ravi A, Ereqat S, Al-Jawabreh A, Abdeen Z, Abu Shamma O, Hall H, Pallen MJ, Nasereddin A. 2019. Metagenomic profiling of ticks: Identification of novel rickettsial genomes and detection of tick-borne canine parvovirus. PLoS neglected tropical diseases 13:e0006805-e0006805.

14. Freeman CN, Scriver L, Neudorf KD, Truelstrup Hansen L, Jamieson RC, Yost CK. 2018. Antimicrobial resistance gene surveillance in the receiving waters of an upgraded wastewater treatment plant. FACETS 3:128-138.
15. Amarasiri M, Takezawa T, Malla B, Furukawa T, Sherchand JB, Haramoto E, Sei K. 2022. Prevalence of antibiotic resistance genes in drinking and environmental water sources of the Kathmandu Valley, Nepal. Front Microbiol 13:894014.
16. Ying GG, He LY, Ying AJ, Zhang QQ, Liu YS, Zhao JL. 2017. China Must Reduce Its Antibiotic Use. Environ Sci Technol 51:1072-1073.
17. Li P, Wu Y, He Y, Zhang B, Huang Y, Yuan Q, Chen Y. 2020. Occurrence and fate of antibiotic residues and antibiotic resistance genes in a reservoir with ecological purification facilities for drinking water sources. Sci Total Environ 707:135276.
18. Luo C, Zhang T, Mustafa MF, Li M, XU S. 2025. Removal efficiency of ARGs in different wastewater treatment plants and their potential risks in effluent. npj Clean Water 8.
19. Bonanno Ferraro G, Bonomo C, Brandtner D, Mancini P, Veneri C, Briancesco R, Coccia AM, Lucentini L, Suffredini E, Bongiorno D, Musso N, Stefani S, La Rosa G. 2024. Characterisation of microbial communities and quantification of antibiotic resistance genes in Italian wastewater treatment plants using 16S rRNA sequencing and digital PCR. Sci Total Environ 933:173217.
20. Sharma VK, Johnson N, Cizmas L, McDonald TJ, Kim H. 2016. A review of the influence of treatment strategies on antibiotic resistant bacteria and antibiotic resistance genes. Chemosphere 150:702-714.
21. Drane K, Sheehan M, Whelan A, Ariel E, Kinobe R. 2024. The Role of Wastewater Treatment Plants in Dissemination of Antibiotic Resistance: Source, Measurement, Removal and Risk Assessment. Antibiotics (Basel) 13.
22. Diggle SP, Whiteley M. 2020. Microbe Profile: Pseudomonas aeruginosa: opportunistic pathogen and lab rat. Microbiology (Reading) 166:30-33.
23. He Y, Li K-X, Wang J-W, Wang W, Fan P-C, Chen H-H, Wang J-J. 2021. Microbial Community Structure of Waste Water Treatment Plants in Different Seasons. Huan jing ke xue= Huanjing kexue / [bian ji, Zhongguo ke xue yuan huan jing ke xue wei yuan hui "Huan jing ke xue" bian ji wei yuan hui] 42:1488-1495.
24. Serletti L, Dutcher L, Degnan KO, Szymczak JE, Cluzet V, David MZ, Cressman L, Glassman LW, Hamilton KW, Centers for Disease C, Prevention Prevention Epicenters P. 2023. Analysis of seasonal variation of antibiotic prescribing for respiratory tract diagnoses in primary care practices. Antimicrob Steward Healthc Epidemiol 3:e147.
25. Velazquez-Meza ME, Galarde-López M, Cornejo-Juárez P, Bobadilla-Del-Valle M, Godoy-Lozano E, Aguilar-Vera E, Carrillo-Quiroz BA, Ponce de León-Garduño A, Velazquez Acosta C, Alpuche-Aranda CM. 2025. Bacterial Communities and Resistance and Virulence Genes in Hospital and Community Wastewater: Metagenomic Analysis. Int J Mol Sci 26.
26. Cai L, Ju F, Zhang T. 2014. Tracking human sewage microbiome in a municipal wastewater treatment plant. Appl Microbiol Biotechnol 98:3317-26.
27. Hendriksen RS, Munk P, Njage P, van Bunnik B, McNally L, Lukjancenko O, Röder T, Nieuwenhuijse D, Pedersen SK, Kjeldgaard J, Kaas RS, Clausen PTLC, Vogt JK, Leekitcharoenphon P, van de Schans MGM, Zuidema T, de Roda Husman AM, Rasmussen S, Petersen B, Bego A, Rees C, Cassar S, Coventry K, Collignon P, Allerberger F, Rahube TO, Oliveira G, Ivanov I, Vuthy Y, Sopheak T, Yost CK, Ke C, Zheng H, Baisheng L, Jiao X, Donado-Godoy P, Coulibaly KJ, Jergović M, Hrenovic J, Karpíšková R, Villacis JE, Legesse M, Eguale T, Heikinheimo A, Malania L, Nitsche A, Brinkmann A, Saba CKS, Kocsis B, Solymosi N, et al. 2019. Global monitoring of antimicrobial resistance based on metagenomics analyses of urban sewage. Nature Communications 10:1124.
28. Dekaboruah E, Suryavanshi MV, Chettri D, Verma AK. 2020. Human microbiome: an academic update on human body site specific surveillance and its possible role. Arch Microbiol 202:2147-2167.

29. Jiao Y-N, Zhou Z-C, Chen T, Wei Y-Y, Zheng J, Gao R-X, Chen H. 2018. Biomarkers of antibiotic resistance genes during seasonal changes in wastewater treatment systems. Environmental pollution (Barking, Essex : 1987) 234:79-87.
30. Lee CR, Lee JH, Park M, Park KS, Bae IK, Kim YB, Cha CJ, Jeong BC, Lee SH. 2017. Biology of Acinetobacter baumannii: Pathogenesis, Antibiotic Resistance Mechanisms, and Prospective Treatment Options. Front Cell Infect Microbiol 7:55.
31. Gómez P, Lozano C, Benito D, Estepa V, Tenorio C, Zarazaga M, Torres C. 2016. Characterization of staphylococci in urban wastewater treatment plants in Spain, with detection of methicillin resistant Staphylococcus aureus ST398. Environ Pollut 212:71-76.
32. Ben Said M, Abbassi MS, Gómez P, Ruiz-Ripa L, Sghaier S, Ibrahim C, Torres C, Hassen A. 2017. Staphylococcus aureus isolated from wastewater treatment plants in Tunisia: occurrence of human and animal associated lineages. J Water Health 15:638-643.
33. Bengtsson-Palme J, Hammarén R, Pal C, Östman M, Björlenius B, Flach CF, Fick J, Kristiansson E, Tysklind M, Larsson DGJ. 2016. Elucidating selection processes for antibiotic resistance in sewage treatment plants using metagenomics. Sci Total Environ 572:697-712.
34. Yang Y, Li B, Ju F, Zhang T. 2013. Exploring variation of antibiotic resistance genes in activated sludge over a four-year period through a metagenomic approach. Environ Sci Technol 47:10197-205.
35. Karkman A, Johnson TA, Lyra C, Stedtfeld RD, Tamminen M, Tiedje JM, Virta M. 2016. High-throughput quantification of antibiotic resistance genes from an urban wastewater treatment plant. FEMS Microbiol Ecol 92.
36. Wen Q, Yang L, Duan R, Chen Z. 2016. Monitoring and evaluation of antibiotic resistance genes in four municipal wastewater treatment plants in Harbin, Northeast China. Environ Pollut 212:34-40.
37. Pärnänen KMM, Narciso-da-Rocha C, Kneis D, Berendonk TU, Cacace D, Do TT, Elpers C, Fatta-Kassinos D, Henriques I, Jaeger T, Karkman A, Martinez JL, Michael SG, Michael-Kordatou I, O'Sullivan K, Rodriguez-Mozaz S, Schwartz T, Sheng H, Sørum H, Stedtfeld RD, Tiedje JM, Giustina SVD, Walsh F, Vaz-Moreira I, Virta M, Manaia CM. 2019. Antibiotic resistance in European wastewater treatment plants mirrors the pattern of clinical antibiotic resistance prevalence. Sci Adv 5:eaau9124.
38. Rumky J, Kruglova A, Repo E. 2022. Fate of antibiotic resistance genes (ARGs) in wastewater treatment plant: Preliminary study on identification before and after ultrasonication. Environ Res 215:114281.
39. Liu Z, Klümper U, Liu Y, Yang Y, Wei Q, Lin JG, Gu JD, Li M. 2019. Metagenomic and metatranscriptomic analyses reveal activity and hosts of antibiotic resistance genes in activated sludge. Environ Int 129:208-220.

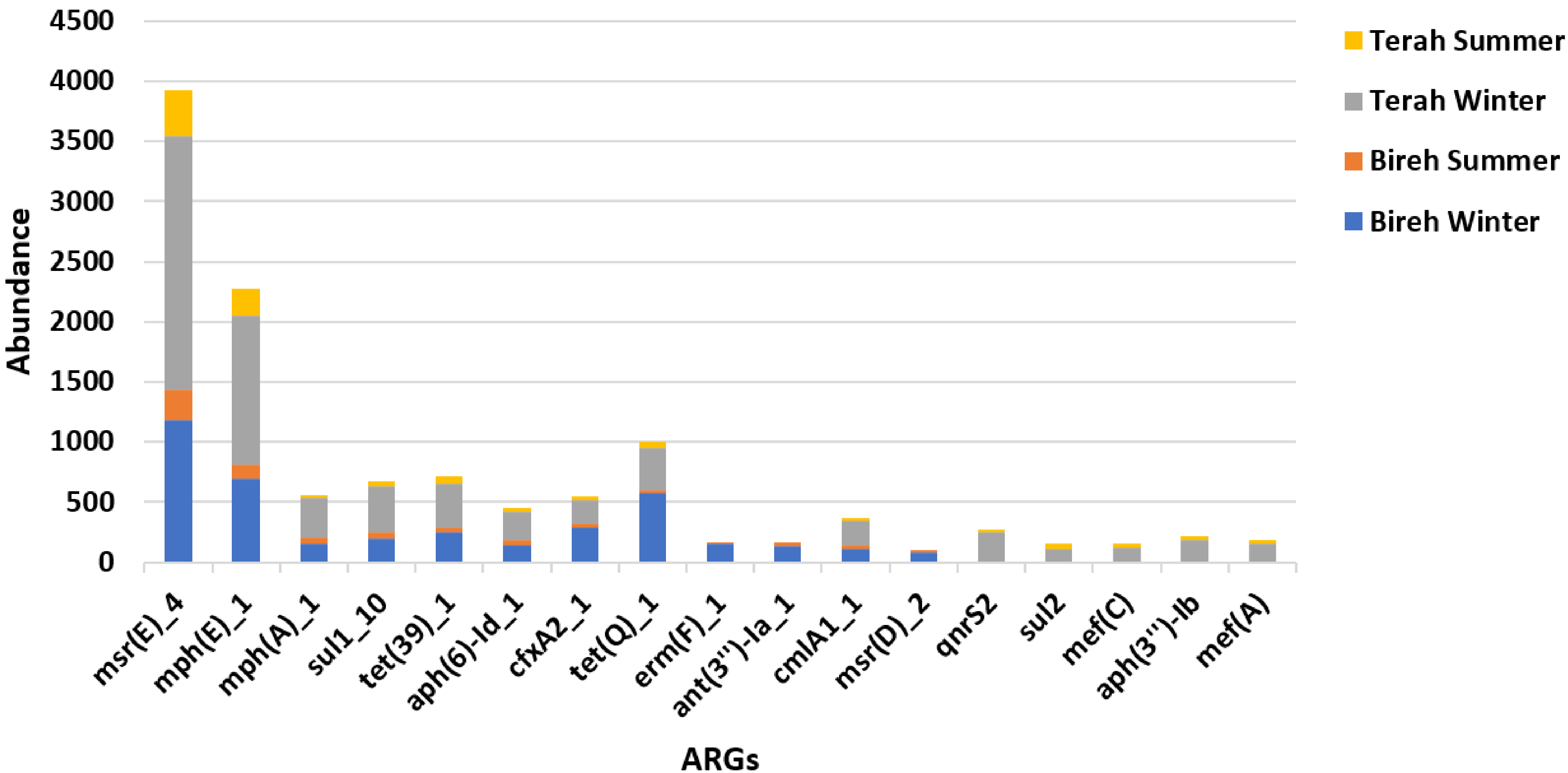

Figure 1: Difference in abundance (No. of reads) of ARGs between winter and summer found in influent samples taken from Al-Bireh and Al-Terah WWTPs

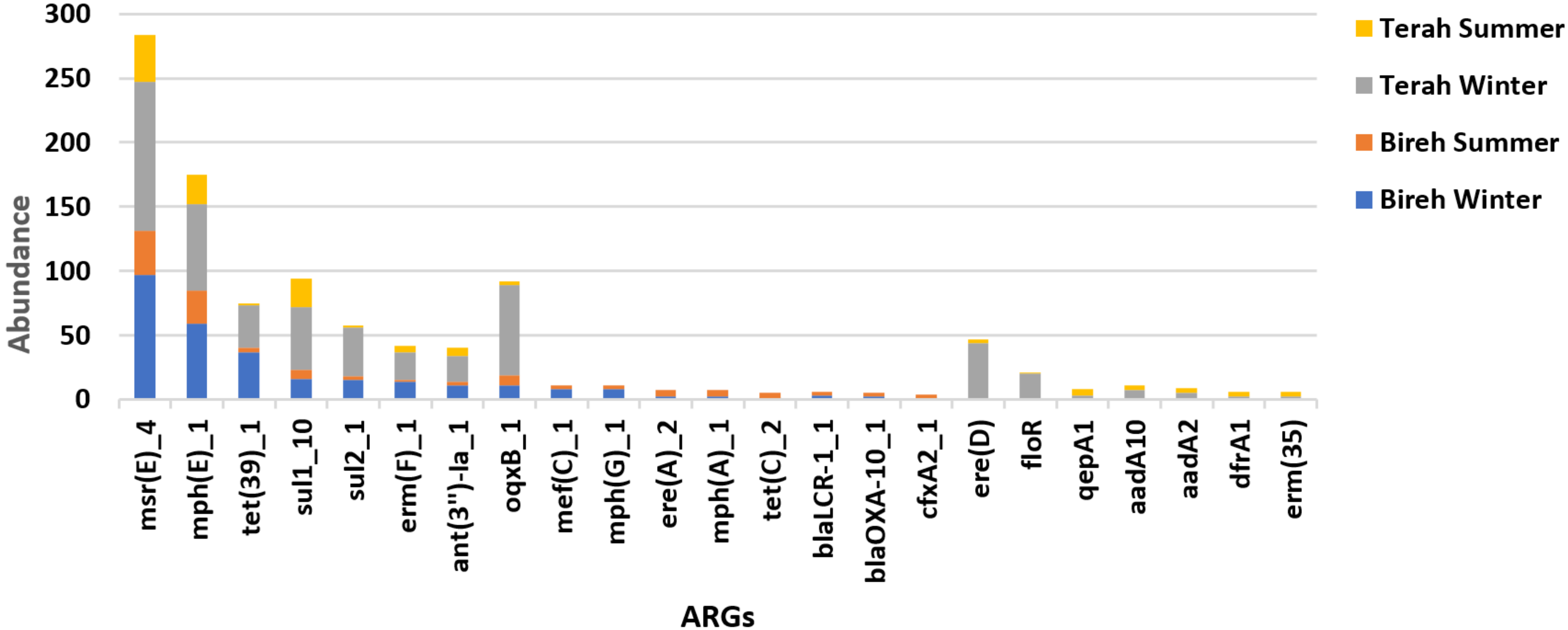

Figure 2: Differences in abundance (No. reads) of ARGs in winter compared to summer in effluent samples from Al-Bireh and Al-Terah WWTPs

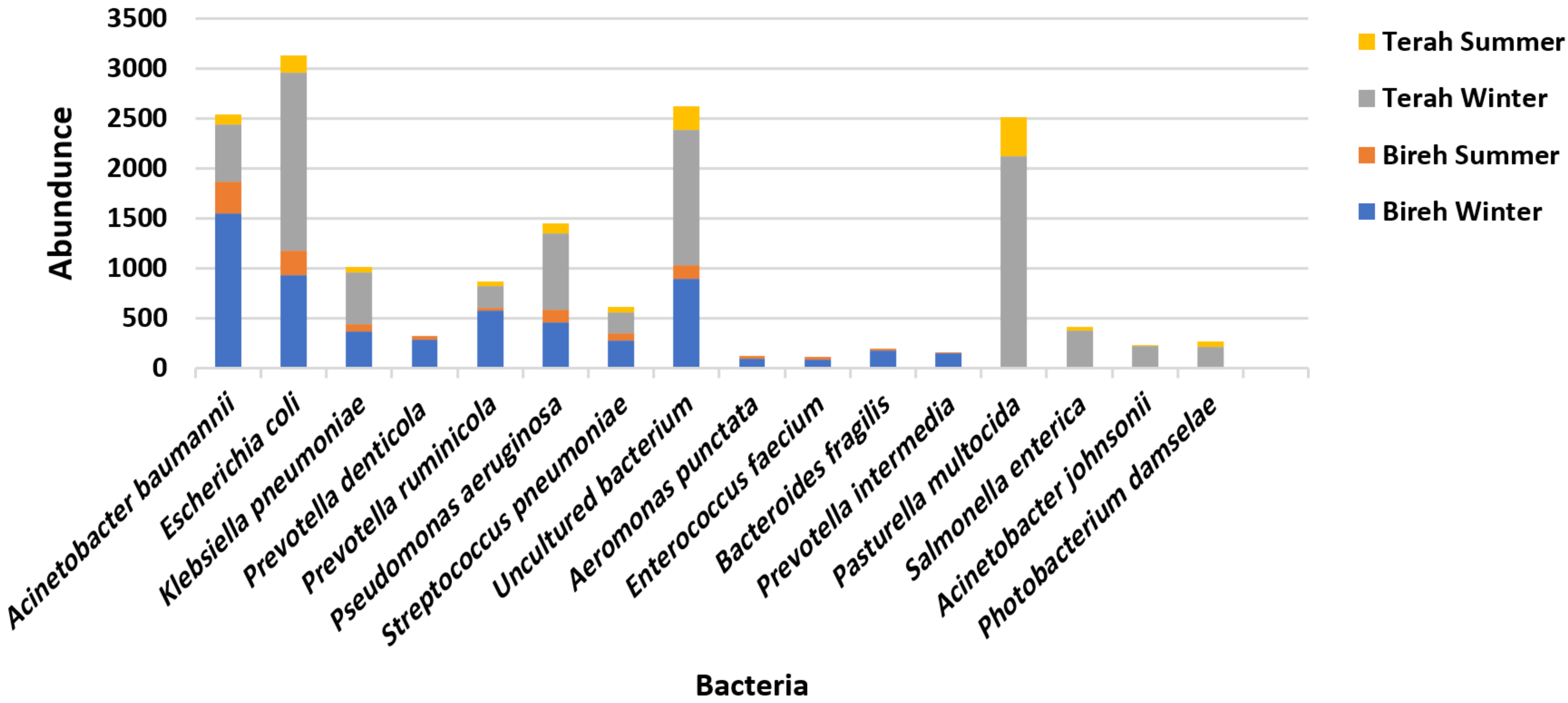

Figure 3: Differences in abundance (No. of reads) of major ARB species between winter and summer in influent samples taken from Al-Bireh and Al-Terah WWTPs

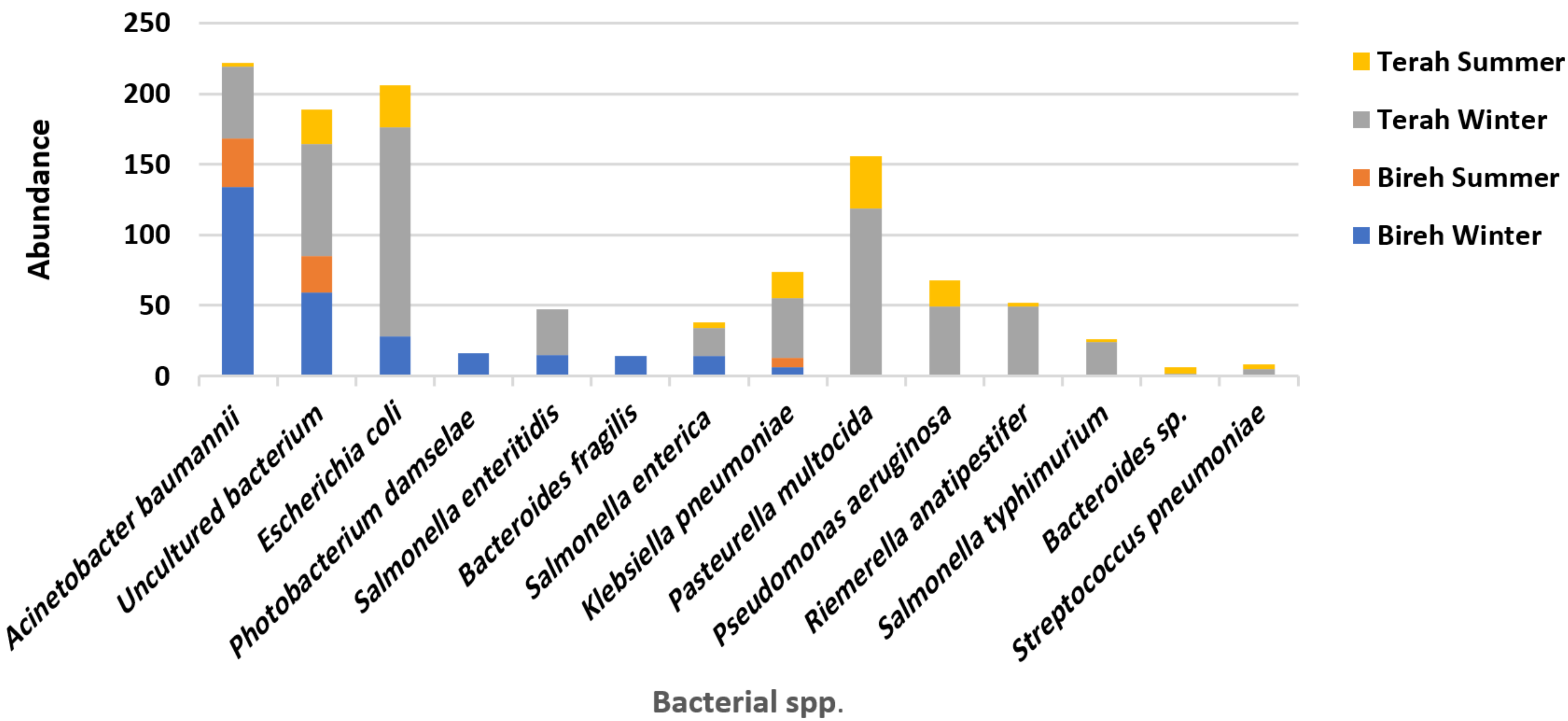

Figure 4: Difference in abundance of the major bacterial species (No. of reads) between winter and summer in effluent samples from Al-Bireh and Al-Terah WWTPs.
