# Supplementary material for: Identification of antibiotic resistance genes and their bacterial hosts in samples collected from wastewater treatment plants in Palestine
Source: Microbiol Spectr. 2026 Sep 18;14(11):e00028-26. doi: 10.1128/spectrum.00028-26 (PMC13729141; doi:10.1128/spectrum.00028-26)
Supplement: Supplementary file 2 [file spectrum.00028-26-s0003.docx]

Appendix Figure 1. (a) The location site of Al-Bireh wastewater treatment plant (AWWTP), north-east of Ramallah city in Palestine ^(1)^. (b) A Google earth map of Al-Tereh WWTP, north-west of Ramallah city in Palestine

(1) Al-Sa'ed, R. & Tomaleh, N. (2012). Performance Evaluation of a Full‐Scale Extended Aeration System in

Al‐Bireh City, Palestine. *CLEAN – Soil, Air, Water*, 40(8), 868-875. https://doi.org/10.1002/clen.201000095
