# Supplementary material for: Identification of antibiotic resistance genes and their bacterial hosts in samples collected from wastewater treatment plants in Palestine
Source: Microbiol Spectr. 2026 Sep 18;14(11):e00028-26. doi: 10.1128/spectrum.00028-26 (PMC13729141; doi:10.1128/spectrum.00028-26)
Supplement: Supplementary file 3 [file spectrum.00028-26-s0004.docx]

Table S1: The removal efficiency of the ten most prevalent ARGs in winter sample (a) and summer sample (b), and the removal efficiency (%) of ten most prevalent bacterial species in summer sample (c) and winter (d)
