# Supplementary material for: Identification of antibiotic resistance genes and their bacterial hosts in samples collected from wastewater treatment plants in Palestine
Source: Microbiol Spectr. 2026 Sep 18;14(11):e00028-26. doi: 10.1128/spectrum.00028-26 (PMC13729141; doi:10.1128/spectrum.00028-26)
Supplement: Supplementary file 4 [file spectrum.00028-26-s0005.docx]

Table S2: ARGs groups revealed by the study based on antibiotic class according to ‘The Comprehensive Antibiotic Resistance Database, CARD’ (a). The ARGs from influent and effluent sample in summer and winter sample at Al-Bireh WWTP (b) and Al-Terh WWTP (c). The bacteria found in the influent and effluent sample in summer and winter sample at Al-Bireh WWTP (d) and Al-Terah WWTP (e).
